# The exocyst complex and Rab5 are required for abscission by localizing ESCRT III subunits to the cytokinetic bridge

**DOI:** 10.1242/jcs.226001

**Published:** 2019-07-17

**Authors:** Harsh Kumar, Kumari Pushpa, Amrita Kumari, Kuldeep Verma, Rajaiah Pergu, Sivaram V. S. Mylavarapu

**Affiliations:** 1 Laboratory of Cellular Dynamics, Regional Centre for Biotechnology, NCR Biotech Science Cluster, 3rd Milestone Faridabad-Gurgaon Expressway, Faridabad, Haryana 121001, India; 2 Manipal Academy of Higher Education, Manipal, Karnataka 576104, India

**Keywords:** Cell division, Cytokinesis, Exocyst complex, ESCRT III, Rab GTPase

## Abstract

Cytokinesis is the final step of cell division following chromosome segregation that generates two daughter cells. The conserved exocyst complex is required for scission of the intercellular cytokinetic bridge, although the molecular mechanisms it employs in this process are unclear. We identify and validate the early endocytic GTPase Rab5 as interacting with the exocyst complex in mammalian cells. Rab5 localizes in the cytokinetic bridge and on the midbody ring in a manner similar to the exocyst complex. Depletion of Rab5 led to delayed abscission. *Caenorhabditis elegans* orthologs of both exocyst complex subunits and Rab5 localize along the cleavage furrow and are required for cytokinesis in early embryos. Cytokinetic cells depleted of either Rab5 or the exocyst subunits Exoc3 and Exoc4 showed impaired deposition of the endosomal sorting complexes required for transport (ESCRT) III subunits CHMP2B and/or CHMP4B near the midbody ring. The study reveals an evolutionarily conserved role for the early endocytic marker Rab5 in cytokinetic abscission. In addition, it uncovers a key requirement of the exocyst and Rab5 for the delivery of components of the membrane-severing ESCRT III machinery to complete cytokinesis.

## Abstract (precis)

**Summary:** The conserved exocytic vesicle-tethering exocyst complex and the endocytic master regulator Rab5 mutually interact and are required to deliver the ESCRT III membrane scission apparatus for completion of cytokinesis.

## INTRODUCTION

Mammalian cells divide with a high degree of fidelity each cell cycle via the process of mitosis to generate two daughter cells with the correct, diploid complement of chromosomes. Mis-regulation of mitosis leads to aneuploidy, a well-established precursor to cancer (Echard, 2012a; Mena et al., 2010). It is therefore imperative for us to elucidate the molecular mechanisms of mitotic regulation in order to understand the basis for asymmetric stem cell division as well as for potential therapeutic intervention in major diseases (Mena et al., 2010). Cytokinesis, the final step of mitosis ensuring the physical separation of daughter cells, is typified by a sequence of complex subcellular events following karyokinesis (nuclear division). These include cytoplasmic furrow ingression mediated by the cortical actomyosin ring at the spindle mid-zone, formation of a dense proteinaceous structure (midbody ring) in the intercellular bridge, trafficking of membrane vesicles to the midbody region and, finally, abscission of the plasma membrane in the bridge leading to separation of the daughter cells (Barr and Gruneberg, 2007; Chen et al., 2012; Echard, 2012a; Schiel and Prekeris, 2013). These events are largely conserved across eukaryotes with a few modifications and are controlled by the coordinated action of various proteins. These include centrosome and midbody ring-associated proteins [e.g. MKLP-1 (also known as KIF23), centriolin, BRUCE and Cep55], kinases (e.g. Plk-1 and Aurora B) and regulators of intracellular traffic like Rab GTPases and their effectors, endosomal sorting complexes required for transport (ESCRT) proteins, the exocyst complex and SNARE proteins (Chen et al., 2012; Elia et al., 2011; Gromley et al., 2003, 2005; Guizetti et al., 2011; Neto and Gould, 2011; Neto et al., 2013b; Schiel et al., 2012).

The exocyst complex is essential for fusion of post-Golgi secretory vesicles (SVs) at sites of exocytic fusion on the plasma membrane (PM) in all eukaryotes (Hsu et al., 1996; Munson and Novick, 2006; Novick et al., 1980; Wang et al., 2002; Wu and Guo, 2015). Based on studies in various eukaryotic systems (Grote et al., 2000; Wu and Guo, 2015), the exocyst, containing eight conserved subunits (named Exoc1 through Exoc8), is believed to tether SVs at sites of fusion on the PM, and also regulates SNARE-mediated membrane fusion (Munson and Novick, 2006; Sivaram et al., 2005; Songer and Munson, 2009). The exocyst complex also interacts with and enables the fusion of Rab11-positive recycling endosomes (REs) at the PM (Fielding et al., 2005; Wu et al., 2005; Zhang et al., 2004). The exocyst complex thus plays pivotal roles in regulating the fusion of both Golgi-derived SVs and REs at the PM. Interestingly, the trafficking of both SVs and REs to the midbody region is required for completion of cytokinesis (Gromley et al., 2005; Hehnly et al., 2012; Schiel et al., 2012; Wilson et al., 2005). In parallel, the localization of the exocyst complex at the midbody ring in mammalian cells is also essential for cytokinesis (Gromley et al., 2005; Neto et al., 2013a). Impeding the localization of the exocyst at the midbody ring by depleting its recruiting factors, such as MKLP-1 and centriolin led to severe delays in abscission (Gromley et al., 2005). These results firmly place the exocyst complex as a central and essential player in mediating the process of abscission. Despite this clear evidence for the requirement of the exocyst, the exact mechanism(s) of exocyst function in cytokinesis remain poorly understood. The exocyst complex is required for cellularization of *Drosophila* embryos (Murthy and Schwarz, 2004) and for anaphase cell elongation in developing *Drosophila* spermatocytes (Giansanti et al., 2015). However, very little is known about the role of the exocyst in other animals in the context of cytokinesis.

In this study, we aimed to gain mechanistic insight into the role of the conserved exocyst complex in cytokinesis. We identify the early endocytic GTPase Rab5 (herein we do not distinguish between the Rab5a and Rab5b forms for mammals) (Gorvel et al., 1991; Rink et al., 2005) as a novel interactor of the exocyst complex in mammalian cells, and show that it localizes at sites of abscission in late cytokinesis similar to what is seen for exocyst complex subunits (Gromley et al., 2005; Pohl and Jentsch, 2008). The exocyst complex colocalized with Rab5, and depletion of Rab5 or of the exocyst subunits Exoc3 or Exoc2, or expression of the Q79L/S34N mutants of Rab5 led to cytokinetic defects. Cytokinetic cells depleted of either Rab5, Exoc3 or Exoc4 showed impaired midbody deposition of CHMP2B and/or CHMP4B, members of the membrane constricting ESCRT III complex, which mediates final cytokinetic bridge abscission (Elia et al., 2011; Guizetti et al., 2011). The *Caenorhabditis elegans* orthologs of Exoc3 (*sec-6*) and Rab5 (*rab-5*) were also found to be enriched along the cleavage furrow and their depletion resulted in cytokinetic defects in early embryos. This work demonstrates an evolutionarily conserved role for these proteins in mediating cytokinesis. We report for the first time the engagement of Rab5 with the exocyst complex, and highlight that these two factors are crucial for recruiting the ESCRT III machinery to mediate membrane abscission in the final step of cytokinesis.

## RESULTS

### Depletion of exocyst complex subunits leads to defective cytokinesis

**Fig. 1.**
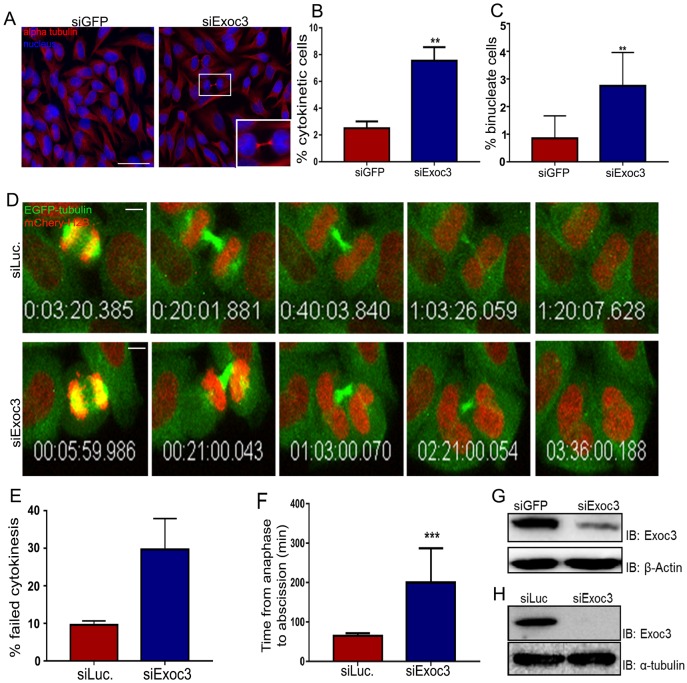
**Exoc3 depletion leads to delayed cytokinesis.** (A) Representative fluorescence micrographs of HeLa cells treated with control or Exoc3 siRNAs, fixed and stained for α-tubulin (red) and chromatin (DAPI). The inset at bottom right shows a magnified image of a cytokinetic cell in the small box. (B,C) Quantification of the percentage of cytokinetic (B) and binucleate cells (C) from >600 cells over three independent experiments, expressed as mean±s.d. (D) Stills from time-lapse confocal movies of a HeLa cell line stably expressing EGFP–α-tubulin (green) and mCherry–H2B (red) treated with siRNAs against control (luciferase, siLuc) and Exoc3 (siExoc3), depicting cells from anaphase onset (first image) to severing of the microtubule bundle in the cytokinetic bridge just prior to membrane abscission (last image). (E,F) Quantification of fraction of cells showing delayed cytokinesis (E; *n*=63 mitotic cells for siLuc and 52 mitotic cells for siExoc3) and cytokinetic timing (F) from time-lapse movies such as shown in D (15 cells across three independent experiments). (G,H) Immunoblots showing Exoc3 depletion for A and D, respectively; β-actin and α-tubulin are shown as loading controls. **P<0.01, ***P<0.001. Scale bars: 75 µm (A); 10 µm (D).

The role of the exocyst complex in cytokinesis has been reported by various groups in the past decade, but mechanistic insight into the cytokinetic function of the complex is missing. We depleted the Exoc3 (Sec6 in worms) subunit of the complex in two mammalian cell lines, namely HeLa (a cervical cancer cell line) and U2OS (a human osteosarcoma cell line) cells via treatment with sequence-specific siRNAs (siExoc3) (Neto et al., 2013a) and immunostained to visualize microtubules (α-tubulin) and chromatin (DAPI) (Fig. 1A). Cells in cytokinesis were identified by the presence of a thin but dense microtubule bundle in the cytoplasmic connection between two cells, representing the late intercellular bridge. Cells depleted of Exoc3 showed an over 3-fold higher cytokinetic index (fraction of cells in cytokinesis) as compared to control knockdown cells (Fig. 1B) as well as a higher fraction of binucleated cells (Fig. 1C), suggesting a delay or arrest in cytokinesis as reported previously for exocyst complex depletion (Gromley et al., 2005; Neto et al., 2013a). In order to further characterize the failure in cytokinesis, we imaged HeLa cells stably expressing histone 2B (H2B)–mCherry and EGFP–α-tubulin (Neumann et al., 2010) and depleted of Exoc3, through confocal fluorescence time-lapse microscopy (Movies 1 and 2; Fig. 1D). We observed a considerable delay in the time taken from anaphase onset to severing of the microtubule bundle near the midbody ring in a significant proportion of cells (∼30%) that had entered mitosis (Fig. 1E), an event that is known to be imminently followed by cytokinetic membrane abscission (Guizetti et al., 2011). We observed similar cytokinetic delays upon Exoc3 depletion in U2OS cells when observing cells through time lapse bright-field imaging, wherein the disappearance of the dark structure in the intercellular bridge (midbody ring) was taken as an indicator of abscission (Movies 3 and 4; Fig. S3D). In both cell types, Exoc3-depleted cells took between 2–3-fold longer to complete cytokinesis as compared to cells treated with control siRNAs (Fig. 1F, Fig. S3E). These data reaffirmed that Exoc3 is essential for mammalian cell abscission. Similar to what was seen for Exoc3, siRNA-mediated depletion of another exocyst subunit Exoc2 also led to a significant cytokinetic defect (∼3-fold) as compared to control depletion (Fig. S3A,B). Taken together, our data suggested that exocyst complex subunits are required for cytokinetic abscission.

### Exoc3 biochemically interacts with Rab5

**Fig. 2.**
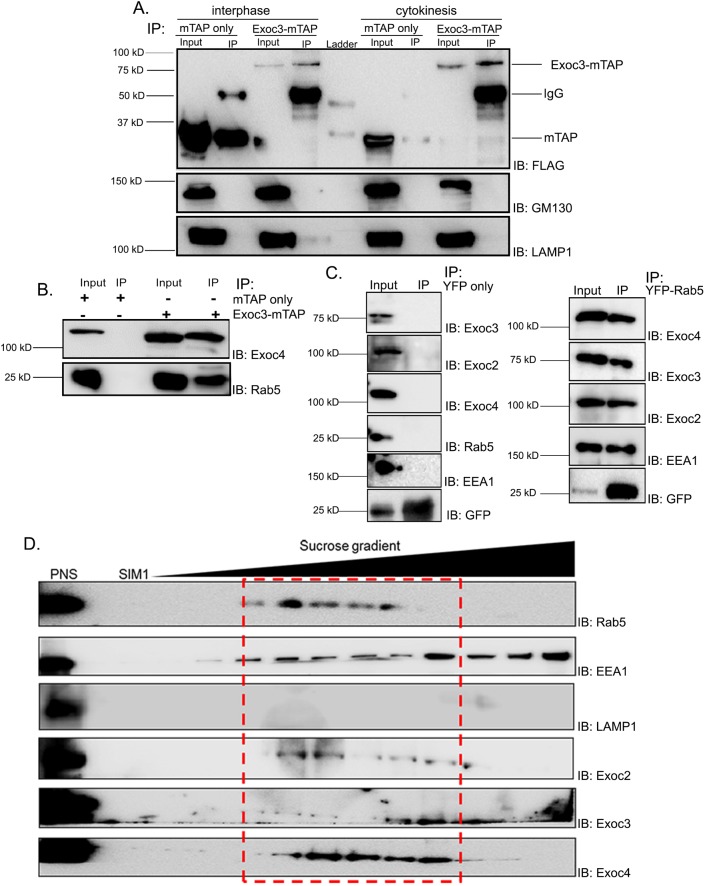
**Exoc3 interacts with early endocytic proteins including Rab5.** (A,B) Immunoblots showing 3× FLAG tag immunoprecipitates (IP) from cytokinetically enriched lysates of U2OS cells expressing the mTAP tag alone or Exoc3–mTAP, and probed for the presence of the indicated proteins (IB). (C) Immunoblots showing YFP tag immunoprecipitates (using the GFP–Trap matrix) from cytokinetically enriched U2OS cells expressing the YFP tag alone or YFP–Rab5 and probed for the presence of the indicated proteins (IB). (D) Immunoblot depicting the subcellular fractions from cytokinetically enriched U2OS cell lysates representing the early endosomal fraction (dashed red box) from a sucrose density gradient experiment and probed for the presence of the indicated proteins (IB).

The literature has reported a functional association of the exocyst complex with endocytosis (Boehm et al., 2017), and an interaction between the exocyst and early endocytic components has been shown in *Drosophila* ovary extracts (Sommer et al., 2005). In mammalian cells, despite the functional association between the exocyst and endocytosis, a biochemical interaction has not been demonstrated. We therefore probed whether the exocyst subunit Exoc3 interacts with the classical early endosome (EE) marker Rab5 (Gorvel et al., 1991; Rink et al., 2005; Stenmark et al., 1994)*.* Towards this aim, we transfected a human Exoc3 construct tagged with a tandem affinity purification tag (Exoc3–mTAP) that included a 3× FLAG tag (Ma et al., 2012) in U2OS cells. We immunoprecipitated Exoc3 from both interphase (asynchronous) and cytokinetically enriched cell lysates by performing anti-FLAG tag immuno-affinity purification (using FLAG-M2 resin). The bait protein Exoc3, its well-documented interactor Exoc4 (Sec8 in worms) (Hsu et al., 1996) and Rab5 were efficiently pulled down with Exoc3–mTAP but not with the tag alone as demonstrated through immunoblotting (Fig. 2A,B). To ensure the specificity of the immunoprecipitation, we probed for markers of other endomembrane compartments, namely the lysosomal marker lysosome-associated membrane glycoprotein 1 (LAMP1) and the cis-Golgi marker GM130 (also known as GOLGA2). Neither of these proteins co-immunoprecipitated with Exoc3–mTAP, demonstrating the specificity of the Rab5 interaction (Fig. 2A). Conversely, we also affinity purified Rab5 from a stable U2OS cell line expressing YFP–Rab5 (Serio et al., 2011), which was able to robustly pull down multiple exocyst complex subunits Exoc2, Exoc3 and Exoc4 (Sec5, Sec6 and Sec8, respectively, in worms) (Fig. 2C). In addition, we performed subcellular fractionation of cytokinetically enriched U2OS cell lysates, EE fractions using sucrose density gradient ultracentrifugation and immunoblotted for early endosomal compartment makers and exocyst complex subunits. Our fractionation experiments showed exocyst complex subunits co-migrating with the fractions representing endosomal compartments, as evident from the presence of endosomal markers Rab5 and EEA1 (Fig. 2D). These biochemical assays demonstrated that the exocyst complex engages with Rab5 and the early endocytic machinery.

### Rab5 is required for cytokinesis

**Fig. 3.**
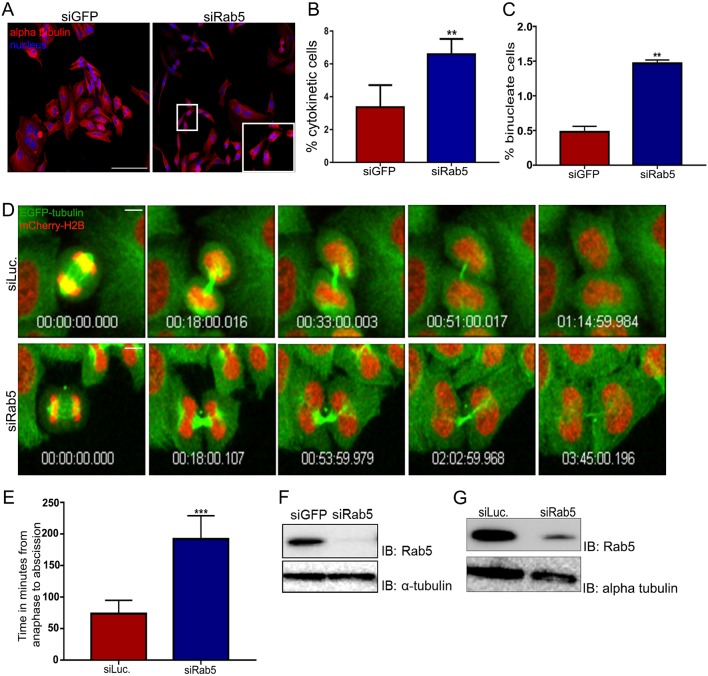
**Rab5 depletion leads to cytokinetic defects.** (A) Representative fluorescence micrographs of HeLa cells treated with control or Rab5 siRNAs, fixed and stained for α-tubulin (red) and chromatin (DAPI, blue). The inset at bottom right shows a magnified image of a cytokinetic cell enclosed by the small box. (B,C) Quantification of the percentage cytokinetic (B) and binucleate cells (C) from >800 cells over three independent experiments, expressed as mean±s.d. (D) Stills from time-lapse confocal movies of a HeLa cell line stably expressing EGFP–α-tubulin (green) and mCherry–H2B (red) treated with siRNAs against control (luciferase, siLuc) and Rab5 (siRab5), depicting cells from anaphase onset (first image) to severing of the microtubule bundle in the cytokinetic bridge just prior to membrane abscission (last image). (E) Quantification of the mean±s.d. cytokinetic timing from time-lapse movies such as shown in D (15 cells across three independent experiments). (F,G) Immunoblots showing Rab5 depletion for A and D, respectively; α-tubulin is shown as a loading control. **P<0.01, ***P<0.001. Scale bars: 75 µm (A); 10 µm (D).

Earlier studies have reported the requirement of the early endocytic pathway in cytokinesis (Chircop et al., 2011; Goss and Toomre, 2008; Kettle et al., 2015); however, a specific cytokinetic role had not been ascribed to Rab5. We therefore asked whether Rab5 functioned in cytokinesis by using sequence-specific siRNA-mediated depletion in HeLa cells and scoring for cytokinetic defects. The depletion of Rab5 using these published siRNA sequences (Chen et al., 2009) was robust, as assessed by western blotting (Fig. 3F,G) followed by densitometric analyses of the immunoblots (Fig. S4D). The cytokinetic index of Rab5-depleted cells (the fraction of cells connected by a thin cytoplasmic intercellular bridge densely packed with microtubules) was nearly two-fold that of control cell populations (Fig. 3A,B), suggesting that Rab5 is required for cells to progress normally through cytokinesis. Similar to what was observed upon Exoc3 depletion, the fraction of binucleate cells also increased significantly (∼3-fold) upon Rab5 depletion (Fig. 3C). Time-lapse imaging of mitotic cells revealed a prolonged interval between anaphase onset and severing of the midbody microtubule bundle, suggesting a significant delay in abscission (Fig. 3D,E), an event that is known to be imminently followed by cytokinetic membrane abscission (Guizetti et al., 2011). In order to assess whether the GTPase activity of Rab5 was required for its function in mediating cytokinesis, we transiently expressed either wild type (WT), the constitutively active GTP-locked form (Q79L mutant) or the constitutively inactive GDP-locked form (S34N mutant) of Rab5 (Mendoza et al., 2013) in HeLa cells (Fig. S6C,D) and scored for cytokinetic defects. Ectopic expression of either of these mutants led to cytokinetic arrest (increased cytokinetic index; Fig. S6A,B). This result suggests that the GTPase activity of Rab5 and the switching between the active and inactive conformations are important for the cytokinetic function of Rab5. Taken together, our results show that there is a requirement for Rab5 in cytokinesis, ascribing a novel function to this well-studied early endocytic GTPase.

### Rab5 and Exoc3 colocalize in the cytokinetic bridge

**Fig. 4.**
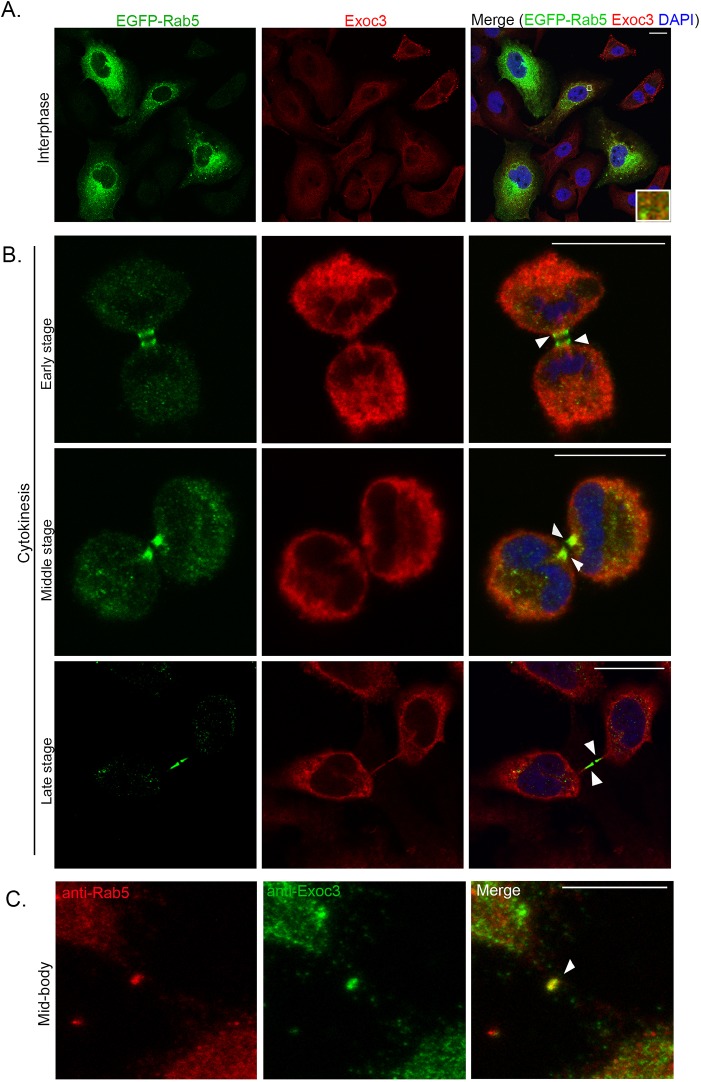
**Rab5 and Exoc3 colocalize during cytokinesis.** (A) Representative confocal micrographs of interphase HeLa cells expressing EGFP–Rab5 immunostained for GFP (Rab5, green) and Exoc3 (red). (B­) Representative confocal micrographs of the cells in A but at cytokinetic stages as indicated. (C) HeLa cells immunostained with antibodies against endogenous Rab5 (red) and Exoc3 (green), showing a cell in late cytokinesis. White arrowheads show the cytokinetic bridge. Scale bars: 10 µm.

Our observations that Rab5 is required for cytokinesis and the known localization of the exocyst complex at the midbody in the cytokinetic bridge (Gromley et al., 2005) prompted us to examine the intracellular localization of Rab5 in the cytokinetic bridge by confocal microscopy. We expressed an enhanced green fluorescent protein (EGFP)-tagged fusion construct of Rab5 (Mendoza et al., 2013) in HeLa cells and immunostained with an antibody against GFP (Camacho et al., 2017; see Fig. 4A for image of interphase cells). We observed discrete and strong localization of EGFP–Rab5 in the cytokinetic bridge on either side of the midbody ring at various stages of cytokinesis, including at the sites of secondary constriction at late stages (Fig. 4B). We also confirmed the cytokinetic localization through immunofluorescence imaging of Rab5 using an antibody against the endogenous protein (Chen et al., 2009, 2014). We observed that Rab5 showed strong localization to the midbody ring in the intercellular bridge, which was also decorated by the exocyst subunit Exoc3 (Fig. 4C). In order to address the apparent difference in localization of endogenous Rab5 (at the midbody ring) and fluorescently tagged Rab5 (flanking the midbody ring), we assessed the late cytokinetic localization of YFP–Rab5 in a stable cell line from another study (Serio et al., 2011), which also mimicked the localization observed with EGFP–Rab5 (Fig. S4A). We authenticated the specificity of the anti-Rab5 antibody used for immunofluorescence by performing siRNA-mediated depletion, which led to a clear loss of signal at the midbody ring (Fig. S4B). It is possible that tagging Rab5 with a small protein such as EGFP/ YFP could impede its localization specifically on the dense midbody ring, despite its robust localization in the flanking regions, in a manner consistent with its requirement for cytokinesis. We observed localization of Exoc3 immunofluorescence at the midbody ring (Fig. 4C) consistent with the literature (Gromley et al., 2005; Neto et al., 2013b). In addition, we observed Exoc3 localization at regions flanking the midbody ring within the bridge both through immunofluorescence against endogenous protein as well as through tagged constructs (Fig. S5A,B).

We quantified the extent of colocalization of Exoc3 and Rab5 in cells by calculating the Pearson's correlation coefficient for their immunofluorescence signals (Collins, 2007). Rab5 and Exoc3 signals colocalized to a small extent in interphase cells (Pearson's coefficient of 0.09±0.06; Fig. 4A). However, colocalization of the two signals increased over 4-fold in cells undergoing cytokinesis (Pearson's coefficient of 0.45±0.19; Fig. 4B). To our knowledge, the above results report the first observation of the localization of Rab5 in the cytokinetic bridge at and near the midbody ring, which supports our functional results showing a role for Rab5 in cytokinesis (Fig. 3A,E). Overall, these microscopy observations also strongly support our biochemical interaction studies and demonstrate that there is a significant association of the exocyst complex with Rab5 in cells, which increases dramatically when cells enter the cytokinetic stage as compared to interphase.

### The exocyst and Rab5 are required for localization of ESCRT III complex subunits at the secondary constriction

**Fig. 5.**
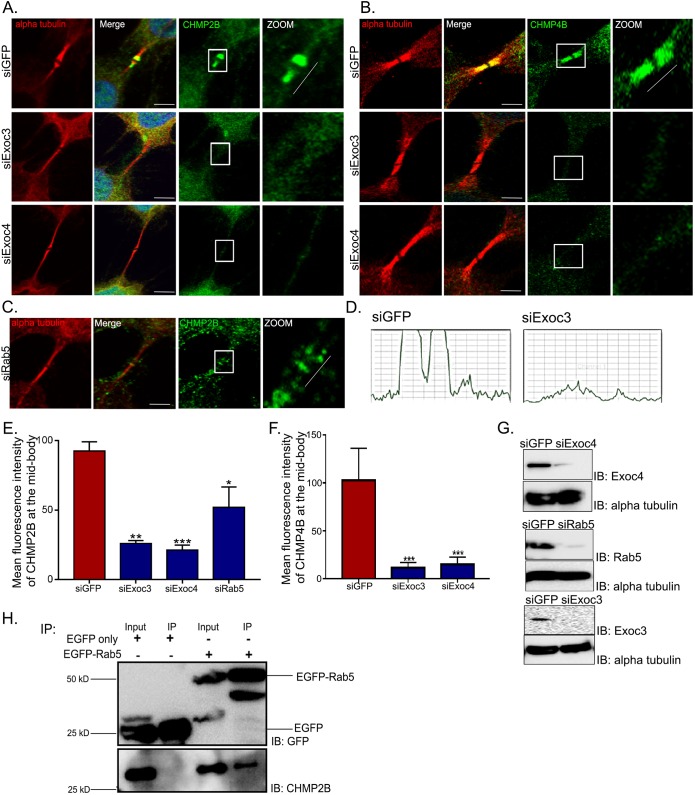
**The exocyst complex and Rab5 deliver ESCRT III subunits to the cytokinetic bridge.** (A–C) Representative confocal micrographs of HeLa cells treated with control (GFP), Exoc3, Exoc4 and Rab5 siRNAs, fixed and stained for α-tubulin (red), CHMP2B (green, A,C), CHMP4B (green, B) and chromatin (DAPI, blue). Scale bars: 10 µm. The area boxed in white is magnified in the image on the right, depicting the midbody ring and secondary constriction region of the cytokinetic bridge. The representative white lines placed beside the cytokinetic bridges (zoom) indicate the corresponding regions within the bridge that were used for line-scan intensity analysis. The lines were of a constant length of 2.32 µm, with the end points coinciding with the secondary constrictions visualized using α-tubulin (microtubule) staining. (D) Representative line-scan profile for CHMP2B intensity (*y*-axis) upon control (siGFP) and Exoc3 (siExoc3) depletion. (E,F) Quantification of mean fluorescence intensity of CHMP2B (E) and CHMP4B (F) at the mid-body region using linescan tool from 20 cells across three independent experiments, shown as graphs with mean±s.d. *P<0.05, **P<0.01, ***P<0.001. (G) Immunoblots showing depletion of the indicated proteins (IB); α-tubulin is shown as a loading control. (H) Immunoblots probing for pulldown of CHMP2B upon immunoprecipitation of EGFP–Rab5 or EGFP. The blot was probed with the respective antibodies as indicated (IB).

We next tried to determine the function of the exocyst complex during cytokinesis. The requirement of the exocyst complex for completion of cytokinesis is known (Gromley et al., 2005; Neto et al., 2013a); however, there are few insights into its molecular mechanism in this process. We observed an abscission defect upon Exoc3 depletion (Fig. 1), consistent with the notion that late cytokinetic events are regulated by the exocyst (Goss and Toomre, 2008; Gromley et al., 2005; Neto et al., 2013a). The penultimate steps in cytokinesis are orchestrated by the ESCRT III protein complex, which localizes on either side of the midbody ring as striated filaments that eventually constrict the membrane and are thought to lead to membrane scission (Elia et al., 2011; Guizetti et al., 2011). We tested whether midbody region recruitment of the ESCRT III complex is governed by the exocyst complex. By using siRNA-mediated depletion of individual exocyst subunits (Exoc3 or Exoc4) in cells, we quantified the levels of the ESCRT III subunits charged multivesicular body proteins 2B and 4B (CHMP2B and CHMP4B) in the intercellular bridges of cytokinetic HeLa cells near secondary constriction sites (called ‘abscission zones’) (Neto and Gould, 2011) using confocal immunofluorescence microscopy. The levels of CHMP2B and CHMP4B were quantified through line-scan analysis in the midbody-proximal region of the bridge (Fig. 5A,B, zoom) and expressed as mean fluorescence intensity (Fig. 5E,F). Consistent with earlier findings (Elia et al., 2011; Guizetti et al., 2011), untreated cells showed strong localization of CHMP2B and CHMP4B on both sides of the midbody ring. In striking contrast, siRNA-mediated depletion of exocyst complex subunits drastically reduced the enrichment of both at these sites, providing the first evidence, to our knowledge, of the exocyst-dependent localization of ESCRT III complex subunits at the abscission zone. Given our observations that Rab5 interacts strongly with the exocyst complex and is also required for cytokinesis (Fig. 2), we similarly probed whether Rab5 affects the abscission zone localization of CHMP2B. Indeed, we also observed a significant reduction in CHMP2B levels at the abscission zone upon depletion of Rab5 from cells (Fig. 5C). In order to assess whether CHMP2B interacted with either Exoc3 or Rab5, we performed co-immunoprecipitation assays with both of these proteins (Exoc3–mTAP or EGFP–Rab5) after transient transfection into U2OS cells and observed a clear interaction of CHMP2B with Rab5 (Fig. 5H). Collectively, these results demonstrate that both the exocyst complex and Rab5 are required for proper localization of the ESCRT III complex to the midbody region, and uncover a vital molecular mechanism that could explain the mechanistic basis for their function in cytokinetic abscission.

**Fig. 6.**
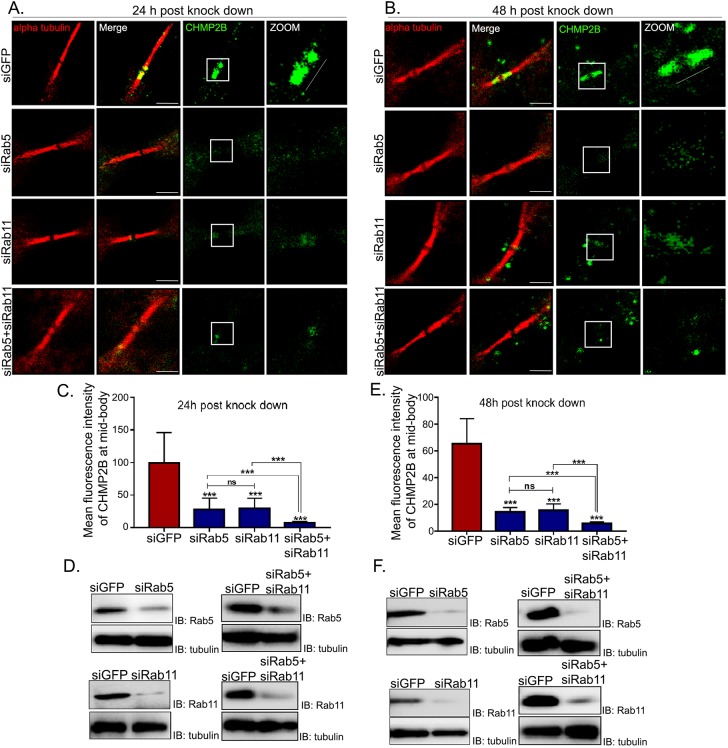
**Rab5 and Rab11 independently recruit CHMP2B at secondary constrictions.** (A,B) Representative confocal micrographs of HeLa cells treated with control (GFP), Rab5, Rab11 or Rab5+Rab11 combined siRNAs for 24 h (A) and 48 h (B), respectively, then fixed and stained for α tubulin (red), CHMP2B (green) and chromatin (DAPI, blue). Scale bars: 10 µm. The area boxed in white is magnified on the right, depicting the midbody ring and secondary constriction region of the cytokinetic bridge. The representative white lines placed beside the cytokinetic bridges (zoom) indicate the corresponding regions within the bridge that were used for line-scan intensity analysis. The lines were of a constant length of 2.32 µm, with the end points coinciding with the secondary constriction visualized using α-tubulin (microtubule) staining. (C,E) Quantification of fluorescence intensity of CHMP2B at the midbody region using the line-scan tool from 20 cells across three independent experiments, shown as graphs with mean±s.d. for A and B, respectively. ***P<0.001, ns, not significant. (D,F) Immunoblots showing depletion of the indicated proteins (IB) for A and B, respectively. α-tubulin is shown as a loading control.

In order to ascertain whether the Rab5 depletion-dependent defect in CHMP2B localization at the secondary constriction was directly caused by an impairment in early endosomal trafficking or an indirect consequence of impaired recycling endosome formation, we performed individual depletion of Rab5 and Rab11 (herein we do not distinguish between the Rab11a and Rab11b form for mammals), as well as combined depletion of both Rab proteins for 48 h and assessed the deposition of CHMP2B at the secondary constrictions. Our siRNA treatments robustly depleted the intended Rab proteins (Fig. 6F). While depletion of either Rab individually reduced the levels of CHMP2B enrichment at the secondary constrictions to ∼20% of control levels, combined depletion clearly led to a significant further reduction of CHMP2B enrichment (Fig. 6B,E). This data strongly suggests that CHMP2B focusing within the bridge is independently carried out by both Rab5 and Rab11, which appear to operate in parallel. We also performed the same experiment using a shorter time of siRNA treatment (24 h, Fig. 6A,C,D). The results from this experiment mirrored those obtained from the 48 h experiment (reduction of enrichment of CHMP2B at secondary constrictions to ∼30% of control levels), confirming that siRNA-mediated Rab5 depletion did not have any major indirect effects on other connected trafficking pathways.

### The exocyst complex is essential for cytokinesis in *Caenorhabditis elegans*

The exocyst complex is conserved across eukaryotes and has been shown to be required for cytokinesis in yeast, mammals and plants (Fendrych et al., 2013; Finger et al., 1998; Gönczy et al., 1999; Gromley et al., 2005; VerPlank and Li, 2005). However, the role of this complex in cytokinesis has been largely uncharacterized in animal systems. With the exception of *Drosophila* spermatocytes, which require the exocyst for cell membrane elongation during anaphase, there is no other study detailing exocyst function during cytokinesis in Animalia (Giansanti et al., 2015). We used *Caenorhabditis elegans*, an excellent model for cell division (Bourdages et al., 2014; Gönczy et al., 1999; Skop et al., 2001; van den Heuvel and Kipreos, 2012), to investigate the function of the exocyst complex in cytokinesis. *C elegans* has one homolog for each of the eight components of the exocyst, namely SEC-3, SEC-5, SEC-6, SEC-8, SEC-10, SEC-15, EXOC-7 and EXOC-8 (Shaye and Greenwald, 2011). We obtained the genetic mutant worms for the Exoc3 ortholog, *sec-6* (tm4536 from NBRP Japan). However, our initial analysis showed that these mutant worms were either embryonic or larval lethal, as also recorded in the WormBase, and could only be maintained as heterozygotes (m/+) that show low brood counts (∼130 per worm with ∼15% larval lethality), but no observable embryonic lethality. We could not observe any mutant (m/m) growing up to adulthood as they die during early larval stages (L1). These phenotypes precluded early embryonic analysis of cell division defects in both heterozygous (m/+) and homozygous (m/m) mutants*.*

**Fig. 7.**
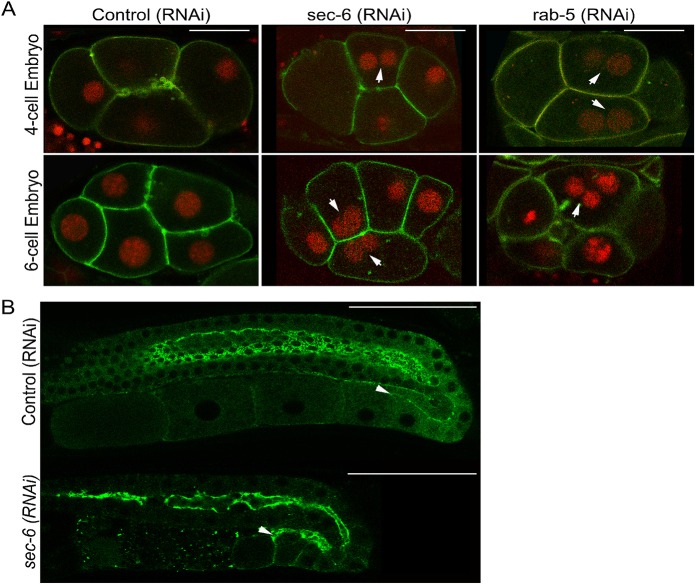
**SEC-6 and RAB-5 are essential** **for cytokiensis in *C. elegans* embryos.** (A) Four-cell (top) and six-cell (bottom) embryos expressing membrane-targeted GFP (PH–GFP) and nuclear-targeted mCherry (H2B–mCherry) from control (vector alone) RNAi, sec-6(RNAi) and rab-5(RNAi) animals. Partial depletion of both *sec-6* and *rab-5* result in multinucleate blastomeres, as indicated by white arrows. (B) SEC-6 is required for cellularization of maturing oocytes. Oocytes remain connected with the gonad via the rachis (marked by white arrowheads), before being completely cellularized during maturation. Cellularization is complete in the most mature oocytes (proximal to the uterus) with control RNAi (top panel). However, upon *sec-6* knockdown, even these oocytes are not completely cellularized (bottom panel). Scale bars: 25 μm.

We therefore performed RNAi-based partial depletion, a standard technique in the field (Conte et al., 2015), to study cell division defects in embryos. Our RNAi resulted in robust knockdown (Fig. S1C) of *sec-6* as indicated by embryonic and larval lethality in the majority of the progeny (F1), consistent with the phenotype observed in the mutant. However, a small percentage of F1 animals that grew up to adulthood and were partially fertile, and were used for this study. We examined the first few divisions in F2 embryos in the absence of SEC-6 (EXOC3) in a transgenic strain expressing PH::GFP (pleckstrin homology domain fused to GFP) and H2B::mCherry (histone 2B fused to mCherry), which decorate the cell membrane and DNA, respectively (Green et al., 2011). As commonly seen in animals, *C. elegans* oocytes are arrested in meiotic prophase and resume meiotic division upon fertilization, extrude two polar bodies and form a mature female pronucleus, which fuses with the male pronucleus from the sperm to give rise to the one-celled zygote. The zygote undergoes mitotic divisions in quick succession producing multicellular embryos (Greenstein, 2005). Failure in cytokinesis is assessed by scoring for the presence of multinuclear blastomeres in the early embryos (Skop et al., 2001). Depletion of *sec-6* (Fig. 7A) resulted in cytokinetic defects in ∼10% (17/154) of *C elegans* embryos, reminiscent of defects earlier reported in yeast and mammalian cells (Dobbelaere and Barral, 2004; Salminen and Novick, 1989; VerPlank and Li, 2005). In addition, *sec-6* depletion also led to failure in polar body extrusion, as indicated by the presence of more than two nuclei in the zygote (Fig. S1A). This was significant because polar body extrusion is a variation of cytokinesis, in which one of the two haploid nuclei formed after meiosis is extruded-out along with very little or no cytoplasm (Maddox et al., 2012). The live imaging data revealed failed cytokinesis at the first meiotic division as a major phenotype (Movies 9 and 10), leading to polar body extrusion defects (Fig. S7). The chromatin of the polar body appears to segregate at anaphase, followed by initiation of membrane ingression between the segregated chromosomes. However, the furrow fails to completely ingress or abscise, and regresses back, leading to a failure of expulsion of the polar body. These data confirm a role for Sec6 in the later stages of cytokinesis, following furrow initiation, in the one-celled *C. elegans* zygote, and are consistent with our observations from mammalian cells, wherein exocyst depletion causes late-stage cytokinesis defects. Just like for sec-6, partial depletion of *sec-8* (*EXOC4* in humans) resulted in embryonic and larval lethality in F2 embryos, as also seen in its mutant, and also in cytokinetic and polar body extrusion defects. We observed cytokinetic defects in multiple early embryonic stages, indicating that the requirement for the exocyst complex may not be stage specific (Fig. 7; Fig. S1B). All of the above results suggest an essential role of the exocyst complex in cytokinesis during *C. elegans* embryogenesis.

During *Drosophila* embryogenesis, the exocyst complex member Sec5 is required for cellularization (Murthy et al., 2010), a process by which a nucleus in a syncytial (multinucleate) tissue gets separated from the syncytium by the closure of the cell membrane (Morgan, 2008)*.* This process is mechanistically very similar to cytokinesis and shares common cellular machinery (Mazumdar and Mazumdar, 2002)*.* This led us to investigate the role of the exocyst complex during cellularization in *C. elegans.* Unlike *Drosophila*, *C. elegans* embryos undergo complete cytokinesis, precluding any study of cellularization during embryogenesis. However, the germline in *C. elegans* is syncytial, wherein each germ cell nucleus is enclosed by membrane from three sides and remains open from one end to a common cytoplasm called the rachis (Amini et al., 2015; Hirsh et al., 1976; Zhou et al., 2013)*.* As the gametes mature, they bud-off from the syncytium and complete cellularization by snapping the intercellular bridges. In wild-type animals, the most mature oocytes present proximal to the uterus are completely cellularized; however, the less-mature ones near the loop region can be seen connected with the rachis (Amini et al., 2015). Components of the centralspindlin complex, namely MKLP1 (ZEN-4) and MgcRacGAP (CYK-4), as well as non-muscle myosin II (NMY-2) and anillins ANI-1 and ANI-2 localize to these intercellular bridges (Amini et al., 2014; Coffman et al., 2016; Lee et al., 2018; Zhou et al., 2013). We used a transgenic line expressing NMY-2::GFP to examine the completion of cellularization in exocyst-depleted worms. We found that the exocyst complex is required for cellularization of maturing oocytes (Fig. S2A). In wild-type animals, oocytes present proximal to the uterus are more mature in comparison to the distal oocytes near the loop region that are just budding out from the syncytium (Lemieux, 1989). As compared to unperturbed worms, *sec-6*-or *sec-8*-depleted animals exhibited a reduced total number of oocytes (Fig. S2B; *n*=37 for control oocytes, 56 for *sec-6* and 36 for *sec-8*) and even the most mature oocytes still remained connected with the rachis, suggesting a delay in oocyte cellularization (Fig. 7B)*.* Taken together, the above results demonstrated that the exocyst complex plays essential roles in cytokinesis in *C. elegans* embryos and oocytes.

### Rab5 is required for cytokinesis in *C. elegans*

Our results in mammalian cells show that Exoc3 biochemically interacts with Rab5 and, similar to Exoc3, Rab5 also has an essential role in cytokinesis. Furthermore, a whole genome RNAi screen in *C. elegans* to identify the genes affecting gonad architecture had reported defects in germ cell cytokinesis in the absence of the *C. elegans* ortholog of Rab5, RAB-5 (Green et al., 2011). These observations led us to test whether *rab-5* plays a role in cytokinesis during early embryogenesis in *C elegans*. Towards this, we performed partial depletion of *rab-5* in the same transgenic strain expressing membrane-targeted GFP with nuclear-targeted mCherry and scored for embryos containing multinuclear blastomeres. Our RNAi resulted in robust knockdown of *rab-5*, leading to embryonic lethality, an earlier reported phenotype (Sönnichsen et al., 2005). As seen in mammalian cells, *rab-5* depletion also led to incomplete cytokinesis in ∼48% (43/88) embryos resulting in multinucleate cells (Fig. 7A)*.* These results strongly suggest that Rab5 is essential for completion of embryonic cytokinesis and that this role is likely to be evolutionarily conserved.

### SEC-6 and RAB-5 localize to sites of active membrane ingression during cleavage divisions

**Fig. 8.**
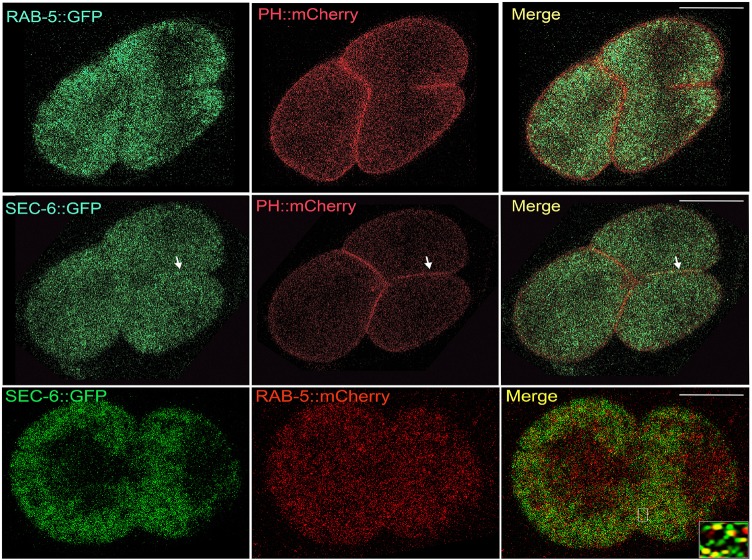
**Both SEC-6 and RAB-5 enrich near the cleavage furrow in *C. elegans* embryos.** Embryos undergoing the second cleavage division expressing either RAB-5::GFP (green, top panel) or SEC-6::GFP (green, middle panel) in addition to PH::mCherry (red) marking the plasma membrane. Both RAB-5 and SEC-6 are enriched along the newly ingressing plasma membrane during cytokinesis. In addition, SEC-6 also decorates the ingressing membrane (white arrows, middle panel). The bottom panel shows a one-cell embryo co-expressing SEC-6::GFP and RAB-5::mCherry undergoing the first cleavage division. The inset depicts a magnified view of the area demarcated by the white rectangle in the merged image showing a section at the cytokinetic plane displaying significant colocalization between SEC-6 and RAB-5. Scale bars: 25 μm.

To gain further understanding into the conservation of the role of the exocyst complex and Rab-5 during cytokinesis, we examined the localization of these proteins in actively dividing *C. elegans* embryos. We generated a fluorescent reporter for SEC-6 by tagging the endogenous protein with eGFP (enhanced GFP) at its C-terminus by using the CRISPR-CAS9 genome editing technique (as described in the Materials and Methods). We further mobilized the membrane-targeted mCherry transgene into this background to enable us to visualize the plasma membrane. Similarly, we used a RAB-5::GFP transgenic line and mobilized the membrane-targeted mCherry transgene in its background. We examined early embryonic cell divisions in these two transgenic lines, and analyzed the localization of SEC-6 and RAB-5 during cytokinesis, using the membrane-targeted mCherry as the reference for the newly ingressing membrane. Both SEC-6 and RAB-5 proteins displayed a punctate cytoplasmic distribution and appeared to be enriched at the cellular cortex near the newly forming membrane (Fig. 8)*.* We observed clear localization of SEC-6::GFP (5/5 embryos) on the ingressing membrane of dividing blastomeres. We also observed enrichment of SEC-6::GFP at the midbody (Movies 5 and 6, Fig. S1D)*.* RAB-5::GFP displayed enrichment along the length of the ingressing membrane, but did not accumulate on the membrane (Fig. 8; Movies 7 and 8)*.* We generated a double-transgenic animal expressing SEC-6::GFP and RAB-5::mCherry and observed colocalization between them along the cytokinetic plane during early embryonic divisions (Fig. 8), consistent with our mammalian cell data (Fig. 4). These results suggest that both SEC-6 and RAB-5 colocalize in *C. elegans* blastomeres and are required for cytokinesis similar to mammalian cells, further strengthening the inference that both these molecules play essential conserved functions in animal cell cytokinesis.

## DISCUSSION

The conserved exocyst complex is required for completion of cytokinesis in mammalian cells (Fededa and Gerlich, 2012; Frémont and Echard, 2018; Gromley et al., 2005; Neto et al., 2013a; Wu and Guo, 2015), as is also the case in multiple other systems and organisms (Glotzer, 2003). The exocyst complex is a key player that associates with both Golgi complex-derived SVs and REs to help fuse them to the plasma membrane (Frémont and Echard, 2018). The trafficking of both SVs and REs to the midbody region of the cytokinetic bridge is essential for abscission (Goss and Toomre, 2008; Gromley et al., 2005; Guizetti et al., 2011). In addition, the exocyst complex prominently decorates the midbody ring in late cytokinesis (Goss and Toomre, 2008; Gromley et al., 2005; Neto et al., 2013b). Preventing the localization of the exocyst complex at this hub of cytokinetic activity by depleting its midbody receptors, like centriolin and MKLP1, leads to delayed cytokinesis (Gromley et al., 2005). The above lines of evidence attest to the central role of the exocyst complex in mediating cytokinesis. However, the exact function of the exocyst and the molecular mechanism(s) for its requirement in cytokinesis were unknown.

Our experiments identify Rab5 as a bona fide interactor of Exoc3 (Fig. 2B,C), an interaction that had thus far not been observed. The exocyst complex also co-migrated with the EE subcellular fraction (Fig. 2D), which was consistent with the association of this complex with EEs. We also observed strong colocalization of Rab5 with the exocyst complex in mammalian cells in the cytokinetic bridge, at the secondary constriction and at the midbody ring using a fluorescently tagged Rab5 construct as well as with a well-characterized antibody against endogenous Rab5 (Fig. 4C). These results corroborated the theory that the exocyst complex engaged with the Rab5-positive endosomal fraction. The over 4-fold increase in colocalization of the Exoc3 with Rab5 in cells undergoing cytokinesis as compared to what was seen in interphase cells (∼43% versus ∼10% colocalization, Fig. 4A,B) indicates that there could be a marked increase in endocytic activity (Montagnac et al., 2008; Schweitzer et al., 2005) and/or increased engagement of the exocyst with the early endocytic machinery as the cell proceeds towards abscission. The exocyst complex is required for the endocytosis of yolk in *Drosophila* oocytes and localizes to clathrin-positive early endocytic pits (Murthy and Schwarz, 2004). Consistent with this, we also observed that the exocyst complex in *C. elegans* is required for the normal uptake of yolk proteins in oocytes, with depletion of the exocyst leading to massive accumulation of yolk in the pseudocoelom of the worm (P.K. and S.V.S.M., unpublished observations). Functionally, the exocyst complex has also been implicated in endocytosis in trypanosomes as well as in human cells (Boehm et al., 2017). Thus, our biochemical and subcellular imaging experiments establish a novel crosstalk between the exocyst complex and the EE marker Rab5 in mammalian cells. To the best of our knowledge, this is the first report of a specific interaction between these two pivotal molecules known to operate at ‘opposite’ incoming and outgoing ends of endomembrane trafficking in the cell.

We observed an ∼2-fold increase in the fraction of cytokinetic cells upon Rab5 depletion (Fig. 3A–C), similar to the cytokinetic arrest observed upon Exoc3 depletion (Fig. 1A–C), which is also consistent with results from a previous report (Yu et al., 2007). This magnitude of increase in the fraction of cytokinetic cells in an asynchronous culture represents a significant arrest in cytokinesis, confirming a novel essential role for Rab5 in mediating cytokinesis. These results were further validated by using the Rab5 ‘constitutively on’ (Q79L) and ‘constitutively off’ (S34N) mutants, which are known to show functional defects in a dominant-negative manner (Galperin and Sorkin, 2003; Stenmark et al., 1994). Indeed, exogenous expression of either of these GTPase mutants also showed an increased cytokinetic arrest in cells (Fig. S6), suggesting that a functional endocytic pathway capable of normally switching between the on and off states of Rab5 is important for cytokinesis. Despite earlier studies on the role of the early endocytic pathway in cytokinesis (Chircop et al., 2011; Goss and Toomre, 2008; Kettle et al., 2015), a specific cytokinetic role had not been assigned to Rab5, which is otherwise very well studied in the context of endocytosis. To the best of our knowledge, our results represent the first study functionally implicating the requirement of Rab5 in completion of cytokinesis.

Our findings in the roundworm *C. elegans*, an established model for cell division studies (Gönczy et al., 1999; Oegema and Hyman, 2006) further confirm the functional role of the exocyst complex and Rab5 in cytokinesis. We observed multinucleate blastomeres in early *C. elegans* embryos upon RNAi-mediated depletion of either the exocyst complex (SEC6) or of Rab5, while normal embryos showed strictly mononucleate blastomeres (Fig. 7). This is consistent with the only other report of a role for the exocyst complex in cytokinesis from a metazoan system, albeit in spermatocytes rather than embryos (Giansanti et al., 2015). Multiple lines of evidence pointed to a direct role for the exocyst in regulating cytokinesis in the embryo, including the cytokinetic localization of SEC-6 in worm embryos at the midbody and near the cytokinetic furrow (Fig. S1D), as well as the cytokinetic phenotypes observed upon *sec-6* RNAi (Figs 7 and 8). This data was consistent with our observations in mammalian cells, which also showed strong cytokinetic localization and function (Figs 1 and 4). Put together with the well-characterized role of the exocyst in cytokinesis in multiple systems (Wu and Guo, 2015), it is tempting to postulate that the exocyst complex has a direct contribution to cytokinesis in *C. elegans* embryos. An additional possible explanation for the observed cytokinetic defects could be through defective extracellular matrix (ECM) and egg shell formation due to impaired secretion (Sato et al., 2008), a phenotype that is reasonable to expect upon exocyst depletion. Defective egg shell formation perturbs its osmotic permeability, causing the membrane of the embryo to swell in the hypotonic uterus, thereby opposing cytokinetic furrow invagination (Kaitna et al., 2002). Thus, it is possible that depletion of the exocyst could indirectly exacerbate cytokinesis failure through perturbation of the osmolarity barrier, which can affect both meiotic and mitotic cytokinesis (Johnston et al., 2006; Kaitna et al., 2002). Deeper investigation is required to delineate the magnitude of osmolarity barrier defects, if any, on embryonic cytokinesis. Nevertheless, our data conclusively demonstrates that the exocyst complex is required for proper completion of cytokinesis in *C. elegans* early embryos.

In addition, oocytes in the *C. elegans* gonad failed to develop fully upon exocyst depletion and showed defects in cellularization in our study (Fig. 7), which is reminiscent of similar defects in cellularization of *Drosophila* embryos observed upon exocyst complex depletion (Murthy et al., 2010). The process of cellularization of the shared oocyte cytoplasm that is continuous with the body rachis in *C. elegans* is topologically analogous to the completion of cytokinesis. Similar to our results implicating a role for RAB-5 in cytokinesis during embryogenesis (Fig. 7), a previous whole-genome RNAi screen to identify the regulators of germline development had revealed a role for RAB-5 in germ cell cytokinesis among other phenotypes (Green et al., 2011). These results together suggest a diverse role for RAB-5 during cytokinesis in different tissue types because, unlike embryos, the germ cells normally undergo incomplete cytokinesis without abscission (Amini et al., 2015). Collectively, our *C. elegans* results report a novel role for the exocyst complex and Rab5 in cytokinesis, and suggest conserved functions for these proteins in this process from invertebrates to vertebrates.

A striking observation in our study is that localization of the ESCRT III subunit CHMP2B was severely impaired in the midbody region upon depleting any one of the exocyst complex subunits Exoc3, Exoc4 or Rab5 (Fig. 5A,B,E,F). We used published and validated siRNA sequences for all of the mammalian cell knockdown experiments. Independent depletion of two exocyst components, Exoc3 (Sec6) and Exoc4 (Sec8) showed similar phenotypes, suggesting that the phenotypes observed were exocyst-mediated and unlikely to be off-target effects. This observation provides the first molecular mechanistic clues to a vital function of the exocyst complex and Rab5 in cytokinesis, namely the delivery of the membrane-constricting ESCRT III machinery to the region of secondary constriction near the midbody ring to ensure successful abscission. The sorting endosome compartment serves as a crucial hub of intracellular vesicular traffic that receives and sends out different types of vesicles, including endosomes of multiple kinds (Naslavsky and Caplan, 2018). Our data suggests that ESCRT III (CHMP2B) recruitment at secondary constrictions is mediated independently through Rab5- and Rab11-dependent (EE and RE, respectively) pathways (Fig. 6). It is possible that ESCRT III complex members are cytoplasmically recruited by Rab5- and exocyst-positive EEs sorted from the EE/sorting endosome compartments and transported to the midbody in preparation for cytokinesis. Indeed, this model could apply to SVs and REs as well; however, further experiments will be needed to determine whether the ESCRT complexes are loaded onto these exocyst-bound vesicles and transported to the midbody. In this study, we have examined the role of Exoc3 and Exoc4, both of which belong to subcomplex 1 of the exocyst complex (Mei and Guo, 2019). However, given that all eight subunits of the mammalian exocyst complex localize at the midbody ring and are required for cytokinetic progression (Gromley et al., 2005; Neto et al., 2013a,b), it is likely that the exocyst holocomplex is required for recruitment of the ESCRT III complex at secondary constrictions.

Closer analysis of our experiments reveals that depletion of the exocyst subunits Exoc3 and Exoc4 led to the most drastic reduction in magnitude of CHMP2B deposition at the midbody, while robust Rab5 depletion led to a slightly milder phenotype (Fig. 5). We speculate that depletion of the exocyst impedes the transport of all three kinds of vesicles (SVs, REs and EEs), while depletion of Rab5 affects only the EEs, which is consistent with the relative extents of the phenotypes. Based on earlier studies, the recruitment of ESCRT III at the secondary constrictions is dependent on the presence of Cep55 at the midbody (Morita et al., 2007), which in turn is dependent on syntaxin-16 (Neto et al., 2013b). Syntaxin-16 is also required for recruitment of the exocyst at the midbody (Neto et al., 2013b). Our results place both the exocyst and Cep55 downstream of Syntaxin-16 in the ESCRT III recruitment pathway. It remains to be seen whether there is a specific order of recruitment of the exocyst and Cep55 at the midbody in preparation for abscission. Multiple models have been proposed to explain why the transport of SVs and REs to the midbody region is required for successful cytokinesis. These include the delivery of extra membrane containing various lipids to enable membrane elongation and/or modulation of midbody membrane fluidity, or enrichment of the midbody-proximal plasma membrane with proteins required for completing cytokinesis (Atilla-Gokcumen et al., 2014; Echard, 2012b; Kouranti et al., 2006). The roles hypothesized for the exocyst complex as a global facilitator of endomembrane fusion originating from various intracellular compartments in the bridge are also further strengthened by the notion that a third class of vesicles (EEs) are recruited to the bridge by the exocyst complex.

In summary, this study reveals a novel interaction between the conserved exocyst complex and the conserved endocytic Rab GTPase Rab5, and uncovers an essential role for Rab5 in mediating cytokinesis in multiple animal systems. It also delineates one specific molecular contribution of both the exocyst and Rab5 – the delivery of components of the membrane-constricting ESCRT III machinery to the cytokinetic bridge for abscission. Our study establishes that the highly conserved exocyst complex is at a pivotal position in the hierarchy of molecules needed to complete cytokinetic abscission, and suggests that this complex could be a central target for further exploration to tease out the mechanisms governing the terminal step of cell division.

## MATERIALS AND METHODS

### Antibodies, DNA and reagents

Primary antibodies against exocyst complex subunits were used as described previously (Neto et al., 2013b). Antibodies for Exoc3 (ab56979 and MA1-2548) were from Abcam, Cambridge, and Thermo Fisher Scientific, respectively; Exoc4 (Sec8, ab13254, 1:1000) were from Abcam, and Exoc2 (Sec5, 12751-1-AP, 1:1500) from Proteintech, USA. Antibodies against Rab5 (HPA003426, 1:1000) (as described previously; Chen et al., 2009), α-tubulin (T6199, 1:2000) and β-actin (A3853, 1:2000) were all from Sigma-Aldrich; anti-GFP (ab6556, 1:2000) and anti-CHMP2B (Bodon et al., 2011; Morita et al., 2010; ab33174, 1:1000 for IB, 1:200 for IF) were from Abcam. Anti-FLAG-M2 (F-1804) antibody was used for immunoblotting as per the manufacturer's recommendation. Anti-mouse-IgG and anti-rabbit-IgG Alexa-Fluor-488-conjugated secondary antibodies (715-545-150, 711-545-152) and anti-mouse-, anti-rabbit-IgG Alexa-Fluor-594-conjugated secondary antibodies (715-585-150, 711-585-152) for immunofluorescence were purchased from Jackson Immunoresearch. Horseradish peroxidase (HRP)-conjugated anti-mouse-IgG (715-035-150) and anti-rabbit-IgG (711-035-152) secondary antibodies for immunoblot analysis were purchased from Jackson Immunoresearch. All secondary antibodies were used at 1:10,000. Exoc3 cDNA was amplified from a human cDNA library and cloned and sequenced in pMTAP-mVenus (kind gift from Dannel McCollum, University of Massachusetts Medical School, Worcester, USA) vector using HindIII (forward primer: 5′-CCTAAGCTTATGAAGGAGACAGACCGGGAGG-3′) and NotI (reverse primer: 5′-GCGGCCGCTCTTGAGCAGCTTGGCCACGTTC-3′) restriction sites. X-tremeGENE HP transfection reagent (Roche Diagnostics) for plasmids and Dharmafect 1 transfection reagent for siRNAs (Dharmacon) were used for transfection. siRNAs were used as described previously: against Luciferase and GFP (Mahale et al., 2016), Exoc3 (Neto et al., 2013a), Sec8 (Sakurai-Yageta et al., 2008) and Rab5 (Chen et al., 2009). GFP-Trap (Chromotek) was used for immunoprecipitation of YFP- or GFP-tagged constructs as earlier previously (Hastoy et al., 2017; Loubéry et al., 2017). EGFP–Rab5 (WT/Q79L/S34N) constructs (Mendoza et al., 2013) were kind gifts from Francisca Bronfman (Pontificia Universidad Católica de Chile, Chile).

### Cell culture, transfection, synchronization and imaging

HeLa cells were purchased from the ECACC (Sigma) and U2OS cells (gift from Stephen J. Doxsey, University of Massachusetts Medical School, Worcester, MA), grown and maintained in DMEM high glucose supplemented with penicillin and streptomycin. HeLa and U2OS cell lines were authenticated towards the beginning of the study. Both cell lines were cultured at 37°C, under 5% CO_2_ and 95% humidity. The H2B–mCherry::EGFP-α tubulin HeLa stable cell line (a gift from Daniel W. Gerlich, Institute of Molecular Biotechnology, The Austrian Academy of Sciences, Austria; Neumann et al., 2010) was cultured in medium supplemented with the antibiotic G418. The YFP–Rab5::RFP–α-tubulin U2OS stable cell line (a gift from Letizia Lanzetti, Instituto di Candiolo, IRCCS, Italy; Serio et al., 2011) was cultured in medium supplemented with neomycin. Cells were transfected with plasmid constructs or siRNAs and assayed at 48 h post transfection. For synchronization, nocodazole was used at 50 ng/ml and 100 ng/ml for HeLa and U2OS cells, respectively. Cells were incubated in nocodazole-containing medium for 14 h, released for 2 h to enrich them in cytokinesis, and fixed or lysed depending upon the assay. For fixed cell imaging, coverslips were imaged with a 40× or 63× objective on a TCS SP8 laser-scanning confocal microscope (Leica, Germany) using confocal/fluorescence modes. Time-lapse microscopy was performed using a 63×1.4 NA oil immersion lens in an environmentally controlled chamber with 5% CO_2_ and 37°C, or as described previously (Mahale et al., 2016) using the same confocal microscope as above.

### Immunofluorescence

Cells were seeded on glass coverslips 24 h prior to siRNA or plasmid transfection. 48 h post transfection media was removed and the cells were washed with phosphate buffered saline and fixed in 3.7% paraformaldehyde at room temperature. Cells were permeabilized and blocked using PBSAT (1× PBS containing 1% BSA and 0.05% Triton X-100). Cells were immunostained with primary and secondary antibodies at room temperature in a humidified chamber for 1 h each. Coverslips were mounted in Prolong Gold/Prolong Diamond antifade mounting reagent containing DAPI (Thermo Fisher Scientific) and allowed to dry and set overnight in the dark.

### Image analysis

For cytokinetic indexing, cells were imaged wtih a 40× magnification objective on an inverted epifluorescence microscope (Leica Microsystems), and the fraction of cytokinetic cells calculated as a percentage of total living cells. Imaging analyses were performed using Leica LASX or ImageJ software modules. Cytokinetic abscission was evaluated from time-lapse series of HeLa stable cells as described above. CHMP2B and CHMP4B intensity at secondary constriction sites was quantified using the line-scan tool of the LASX software. The degree of colocalization between Rab5 and Exoc3 was quantified. To quantify colocalization, a randomly chosen field of cells was selected as a region of interest and analysis was carried using ImageJ-Fiji (http://fiji.sc/Fiji; Collins, 2007). The Pearson's correlation coefficient (*r*) between the two fluorescent signals was calculated from the confocal *z*-stacks. Values represent the mean±s.d. from each image.

### Immunoprecipitation

U2OS cells were transfected with Exoc3 plasmid constructs. At 36 h after transfection, cells were treated with nocodazole for 14 h, washed and released into nocodazole-free medium for 2 h. Cytokinetically enriched cells were harvested and lysed in immunoprecipitation (IP) buffer containing 50 mM HEPES, 150 mM NaCl, 0.1% NP-40 and supplemented with HALT protease and phosphatase inhibitor cocktail (Thermo Fisher Scientific). All steps from lysis onwards were performed on ice. Cells were briefly sonicated for 50 s at 50% amplitude in five pulses, and centrifuged at 15,000 ***g*** (12,000 rpm) at 4°C for 30 min. The supernatant thus obtained (input) was stored on ice. FLAG M2 affinity gel (Sigma-Aldrich) was washed three times in the same IP buffer. Then, 2 mg of input (total protein) was taken for IP and incubated with affinity gel overnight in a cold room on a nutator with gentle rotation. Following incubation, the sample was centrifuged at 200 ***g*** (1500 rpm) for 10 min, the unbound supernatant decanted and the bound affinity gel washed three times in IP buffer. The bound protein was eluted by boiling the affinity gel in 2× Laemlli buffer at 95°C for 10 min and samples were immunoblotted.

### Subcellular fractionation of endosomes by ultracentrifugation

Early endosomes were purified as described previously (Gorvel et al., 1991; Urbanska et al., 2011) with some modifications. Briefly, U2OS cells were resuspended in cold cells breaking buffer (250 mM sucrose, 3 mM imidazole, pH 7.4, protease inhibitor tablet) and lysed on ice using a 5 ml syringe needle. The lysate was centrifuged and the post nuclear supernatant (PNS) was obtained. The PNS was then adjusted to 40.6% sucrose and loaded at the bottom of a 5 ml ultracentrifuge tube compatible with a P40ST rotor (Hitachi Koki Co. Ltd., Japan). The PNS was overlaid sequentially with 35%, 25% and 10% sucrose in 3 mM imidazole, pH 7.4. The gradient was centrifuged at 100,000 ***g*** for 2 h at 4°C. The early endosome fraction was collected from the 35%–25% interface of the gradient and equal volumes of fractions were subjected to SDS-PAGE followed by immunoblotting.

### Immunoblottting

Except for IP-based samples, all other samples for immunoblotting were lysed in 1× RIPA lysis buffer and protein concentration was estimated using the bicinchoninic acid (BCA) assay method using a kit (Thermo Scientific). 20 µg of cell lysates were loaded on a 10% SDS-PAGE gel and electrophoresis was performed before transferring the resolved proteins onto Immobilon-P PVDF membrane (Millipore). Blocking of the membrane was performed in 1× TBST containing either 5% defatted skimmed milk (HiMedia) or 5% BSA (Sigma-Aldrich) followed by overnight incubation in primary antibody at 4°C. Membranes were washed with 1× TBST for 2 h and incubated with HRP-conjugated secondary antibody for 1 h at room temperature. Blots were washed with 1× TBST for 3 h, and ECL substrate (Luminata Forte, Millipore) was added to develop signal. Blot images were captured in an Image Quant LAS 4000 series machine (GE Healthcare Life Sciences).

### Statistical analysis

Unpaired Student's *t*-test was applied on the datasets using Graphpad Prism7 software (San Diego, CA). Graphs and statistical parameters were generated from at least three independent experiments.

### *Caenorhabditis elegans* culture, transgenics, RNAi and imaging

#### Strains

*C. elegans* strains were cultured as per standard practice (Brenner, 1974). The Bristol N2 strain was used as the wild type. The transgenic strains were cultured at 25°C. Double-transgenic strains were generated using standard genetic techniques. The strains used in this study are listed in Table S1. Several of the strains were sourced from *Caenorhabditis* Genetics Center (CGC), Minnesota, USA and the National Bioresource Project (NBRP), Japan.

#### Construction of transgene

The services of Nemametrix Inc. (formerly Knudra Transgenics), USA were used to generate a CRISPR-based EGFP::SBP knock-in at the *sec-6* native locus. Briefly, the CRISPR-select method (Dickinson et al., 2015) was used to knock-in eGFP::SBP at the C-terminus of the *sec-6* native locus. Two guide RNAs, sgRNA1 (5′-AAATTTCCGAGCAAATGAAG-3′) and sgRNA2 (5′-ACAGCAGAAAGCAATTCAGC-3′) were designed to target the C-terminus of *sec-6*. The donor homology plasmid was made using a 757 base pair (bp) left homology arm and a 500 bp right homology arm of the *sec­6* gene flanking a GFP::SBP sequence with a floxed SEC cassette in an intron within the GFP. Injections were performed with standard CRISPR-like (Dickinson et al., 2015) mix in three sets of ten animals each. Injected animals were screened for roller movement on HygR plates (250 µg/ml final concentration). Survivor rollers negative for array markers were separated on regular plates and heat shocked. Wild-type animals were harvested and confirmed by PCR for insertion at the correct site.

#### Microscopy

The embryos were osmo-sensitive (slight shrinkage of membrane observed) upon *sec-6* and *rab-5* knockdown when put in egg buffer (25 mm HEPES pH 7.3, 118 mM NaCl, 48 mM KCl, 2 mM CaCl_2_ and 2 mM MgCl_2_). Imaging was performed *in utero* by anesthetizing the animals for 15 min in M9 buffer containing 0.1% tricane and 0.01% levamizole and mounting on 2% agar pads (McCarter et al., 1999). For localization experiments, animals were dissected in egg buffer on coverslips, mounted on 2% agar pads and sealed with paraffin wax. Images were acquired using a Leica TCS SP8 laser scanning confocal microscope with hybrid detectors (Leica Microsystems Inc.) using a 63× oil objective. The images were processed for clarity of presentation using the LAS X software (Leica Microsystems Inc.) and Photoshop (Adobe Systems).

#### RNA interference

We generated the RNAi constructs for sec-6 and sec-8 by cloning 500 bp of the respective cDNA sequences (*sec-6* forward, 5′-TCTAAGCTTTGGACGTTGATGTGGAAGAG-3′; *sec-6* reverse, 5′-TCTAAGCTTGAACTTCGGCCAGCAATTCG-3′; *sec-8* forward, 5′-TCTCCCGGGCTAGAAGGCATCGACCATTG-3′; *sec-8* reverse, 5′-TCTCCCGGGTCCACTCGTGATAATCGTCC-3′) in the RNAi vector pSV2 (gift from Kuppuswamy Subramaniam, Indian Institute of Technology, Madras, India) at the EcoRV site. The RNAi construct for Rab-5 was sourced from the Ahringer RNAi library (gift from Kuppuswamy Subramaniam). RNAi was performed by feeding method as per set protocols (Timmons and Fire, 1998) except that the final induced culture was concentrated 50-fold before spotting the RNAi plates and used fresh without incubating any further. For *sec-6* and *sec-8*, young adults were kept on the RNAi plate and F1 progeny were examined. For *rab-5*, a mix of L1–L2 stage worms was put on the plate and examined 48 h post RNAi.

## Supplementary Material

Supplementary information
