# Supplementary material for: The exocyst complex and Rab5 are required for abscission by localizing ESCRT III subunits to the cytokinetic bridge
Source: J Cell Sci. 2019 Jul 17;132(14):jcs226001. doi: 10.1242/jcs.226001 (PMC6679584; doi:10.1242/jcs.226001)
Supplement: Supplementary information [file joces-132-226001-s1.pdf]

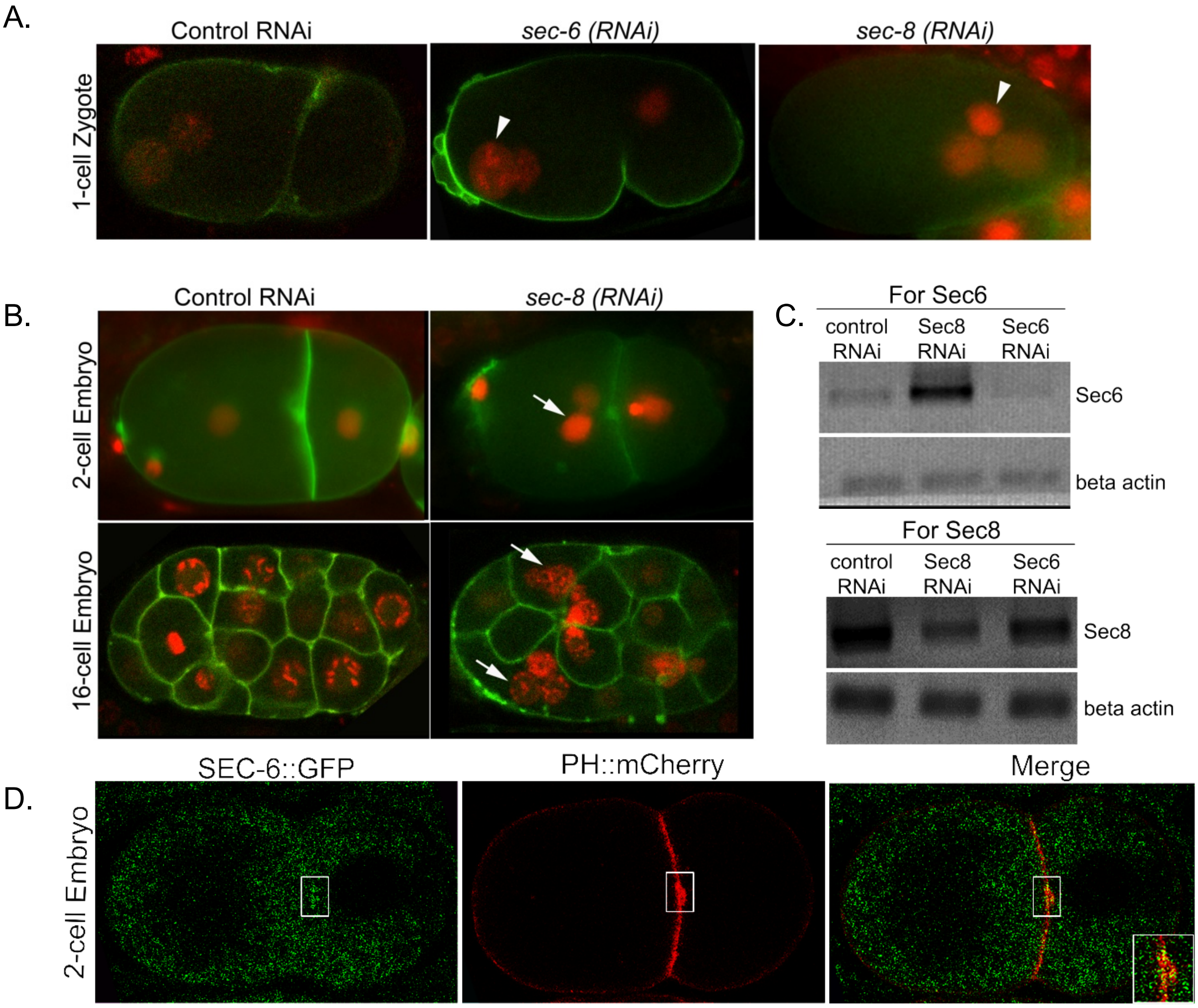

Figure S1: **Exocyst complex subunits are required for cytokinesis in *C elegans* embryos:** **A**. 1-cell zygote expressing membrane GFP (PH-GFP) and nuclear mCherry (H2B-mCherry) from control RNAi, *sec-6(RNAi)* and *sec-8(RNAi)* animals. In wild type conditions, a polar body is extruded before the male and female haploid pronuclei meet such that the 1-cell zygote either contains two haploid pronuclei or one diploid nucleus. Partial depletion of both *sec-6* and *sec-8* resulted in the zygote retaining one or both polar bodies displaying more than two nuclei at the 1-cell stage marked with white arrowheads. **B.** Exocyst component SEC-8 is also essential to complete cytokinesis in *C elegans* embryos. Shown here are 2-cell (top) and 16-cell (bottom) stage embryos displaying multinucleate blastomeres depicted by white arrows. **C.** Semi-quantitative RT-PCR gels showing specific reduction in levels of indicated genes as compared to the β-actin control. Increased mRNA expression of sec6 upon RNAi of sec8 (top panel, middle lane) was observed, perhaps as a compensatory transcriptional mechanism. **D.** Exocyst component SEC-6 localizes to the midbody. A two cell stage *C elegans* embryo expressing SEC-6::GFP and membrane marker PH::mCherry visualized right after the first

Journal of Cell Science • Supplementary information

cleavage division shows localization of SEC-6 at the midbody. The midbody region is enclosed in a white rectangle and the same area is zoomed in the merged panel.

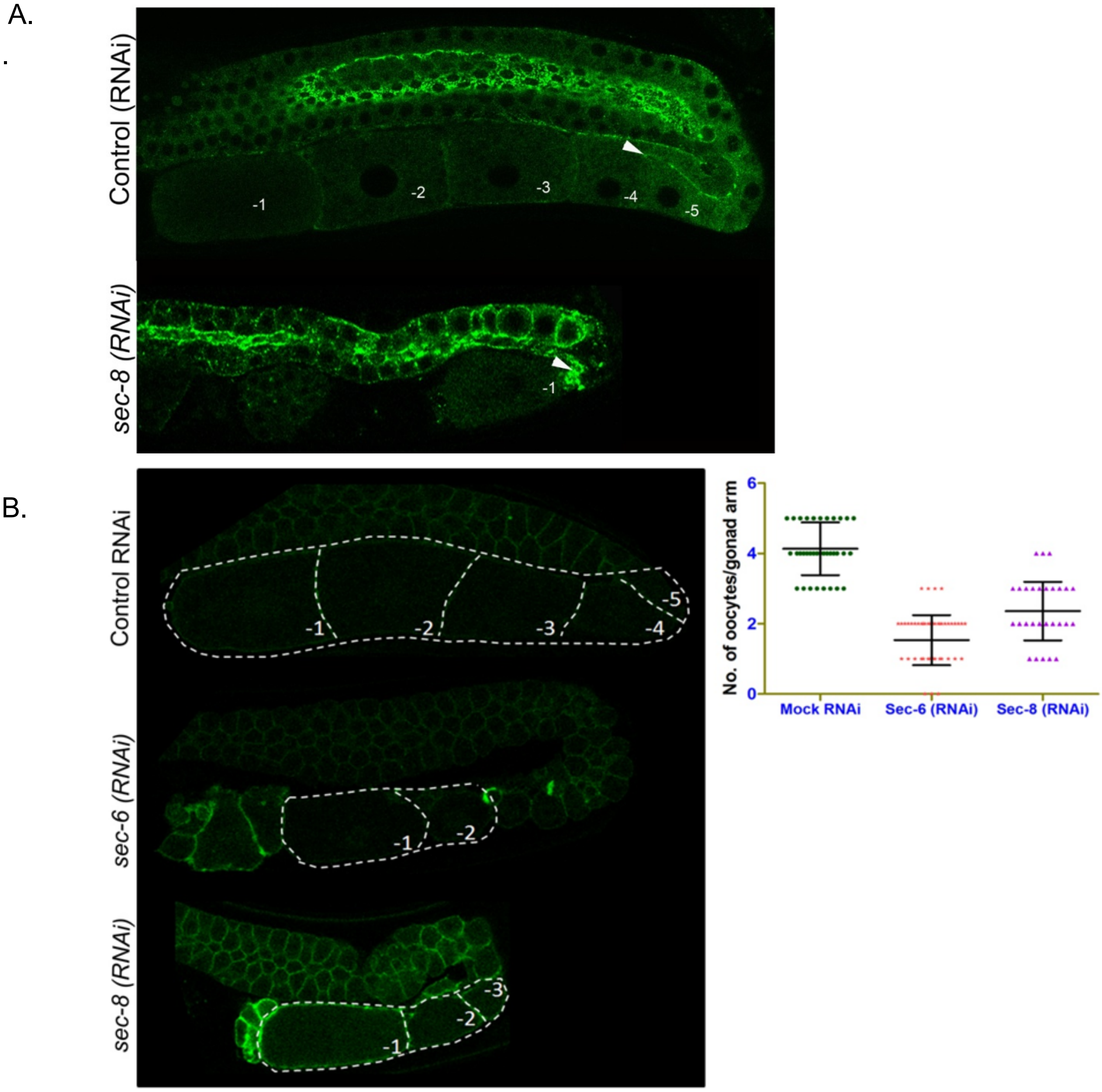

Figure S2: A. Exocyst complex component SEC-8 is required for oocyte cellularization in *C elegans*. Oocytes remain connected with the gonad via the rachis (marked by white arrowhead) initially during maturation. Cellularization is completed in the mature oocytes (-1 to -3) in control RNAi (top panel); however, upon *sec-8* knockdown even the penultimate (-1) oocyte is not completely cellularized and remains connected to the rachis (bottom panel). **B. Exocyst complex components are required for proper oogenesis in *C elegans*.** Control RNAi animals (top image) displayed the normal number of oocytes in the gonad, however RNAi mediated depletion of Exocyst complex components sec-6 (middle image) or sec-8

(bottom image) led to significant reduction in oocyte numbers, indicating defects or delay in oocyte maturation.

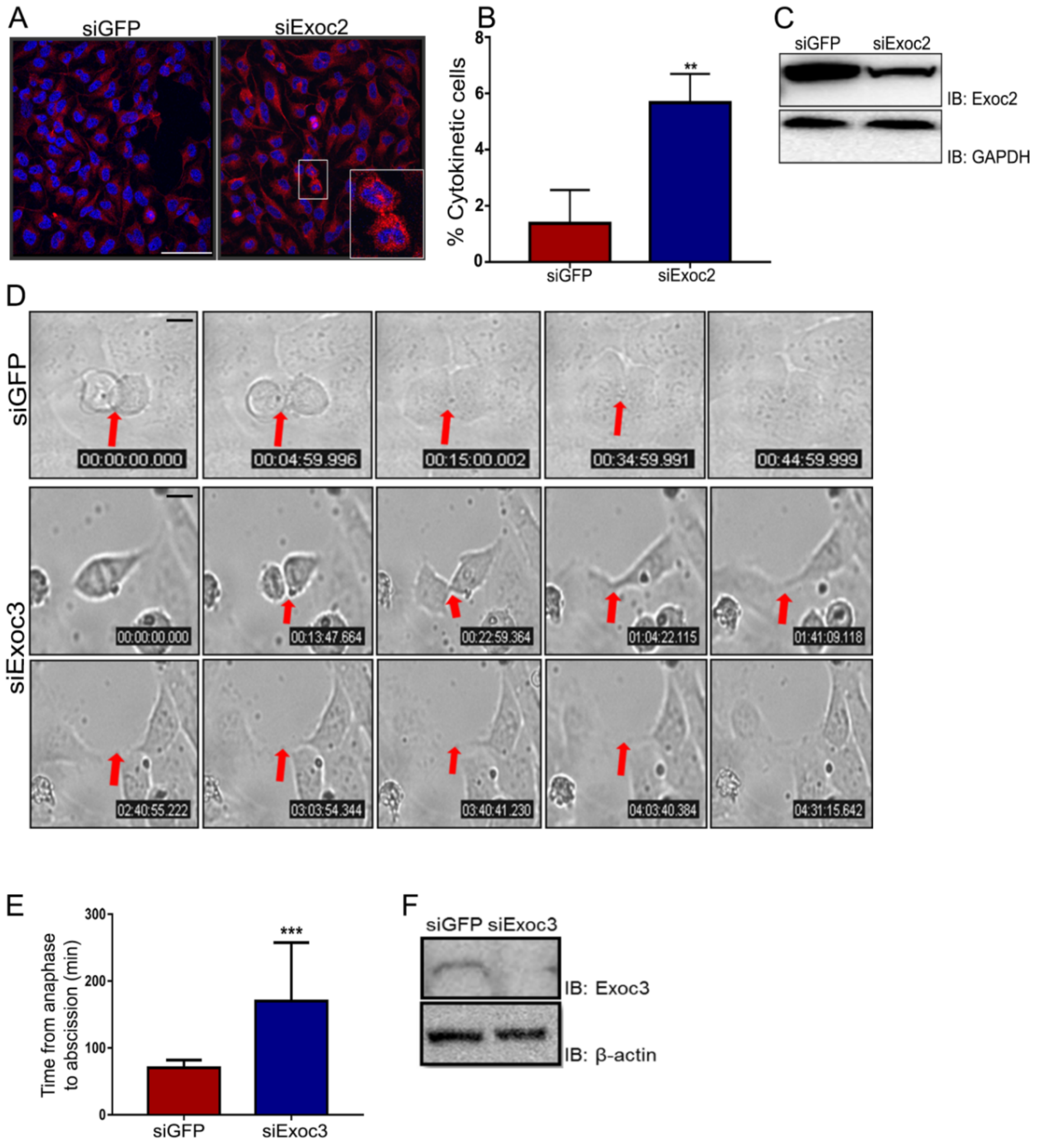

Figure S3: Depletion of Exocyst subunits Exoc2 and Exoc3 leads to cytokinetic defects. **A**. Representative fluorescence micrographs of HeLa cells treated with control or Exoc2 siRNAs, fixed and stained for α tubulin (red) and chromatin (DAPI). Scale bar: 75 µm, inset at bottom right shows zoomed image of a cytokinetic cell enclosed in small box. **B.** Quantification of percent cytokinetic cells from >600 cells over three independent experiments expressed as mean ± S.D. (*P< 0.05). **C.** Immunoblots showing Exoc2 depletion, GAPDH = loading control. **D.** Time-lapse stills (brightfield) from U2OS cells treated with siRNAs against control (GFP) and Exoc3 (siExoc3). Red arrows mark the position of the midbody ring. **E.** Quantification of cytokinetic timing from time-lapse movies such as shown in D (10 cells

Journal of Cell Science • Supplementary information

across three independent experiments). **F**. Immunoblot shows Exoc3 depletion, β actin = loading control.

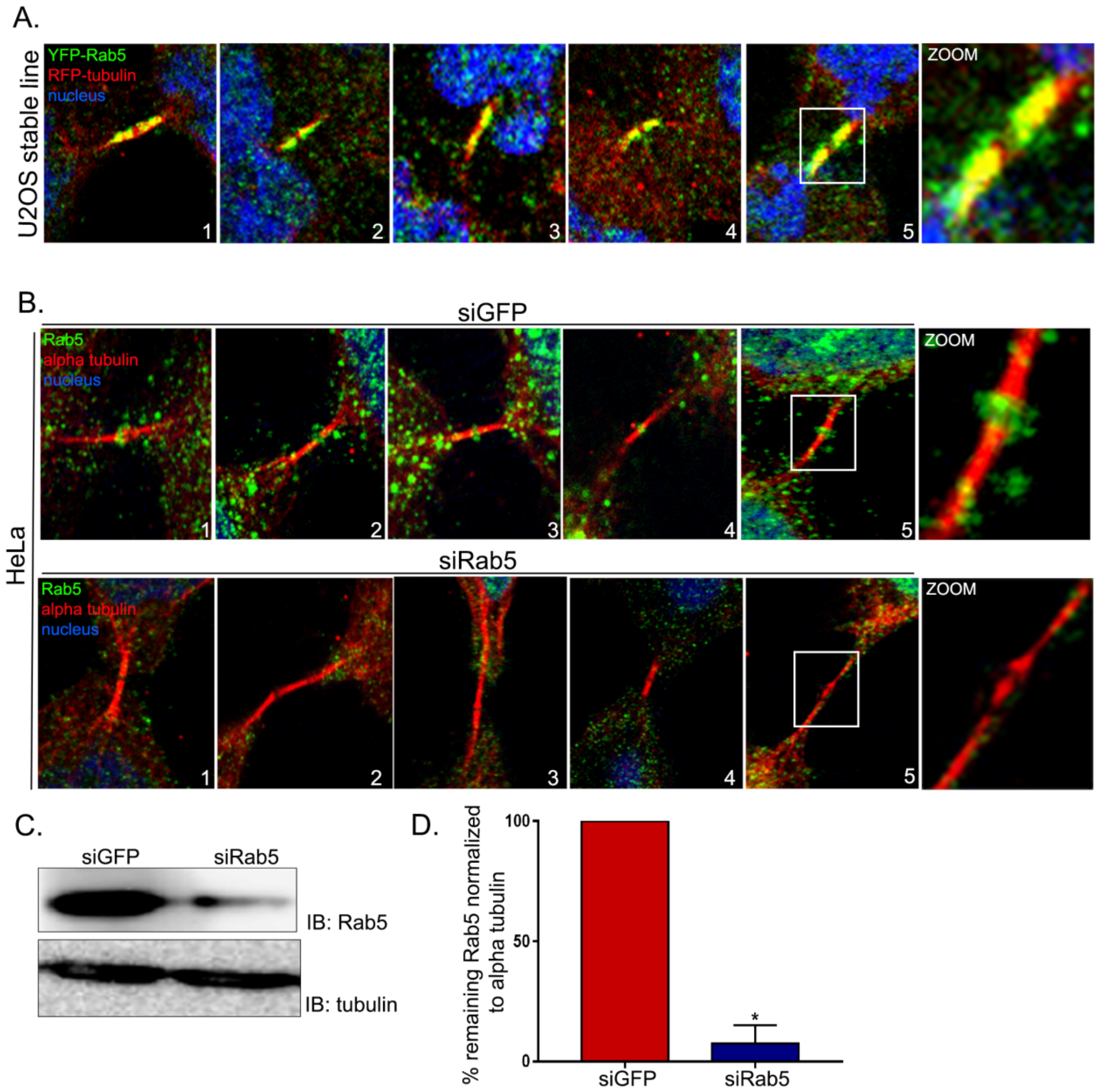

Figure S4: Rab5 localization in the cytokinetic bridge. A. Representative confocal micrographs of 5 independent cells in late cytokinesis of a YFP-Rab5 expressing stable U2OS cell line (Serio et al, 2011). Green = YFP-Rab5 (immunostained with an anti-GFP antibody, green) in the cytokinetic bridge at the regions flanking the midbody ring. Red = RFP-tubulin (microtubules) stained using anti-tubulin antibody. Blue = DAPI (chromatin). The images are merged micrographs of all 3 fluorescence channels. **B.** Confocal micrographs of 5 HeLa cells each treated with control or anti Rab5 siRNA as indicated and immunostained for Rab5 (green), tubulin (red) and chromatin (DAPI, blue). The disappearance of the Rab5 signal at the midbody ring confirmed the specificity of the antibody. **C.** Immunoblot demonstrating the depletion of Rab5. **D.** Densitometry showing ~90% depletion of Rab5 upon siRNA treatment. HeLa cells

Journal of Cell Science • Supplementary information

were transfected with siRNAs against Rab5. The transfected cell lysate was immunoblotted to ascertain the level of corresponding protein depletion.

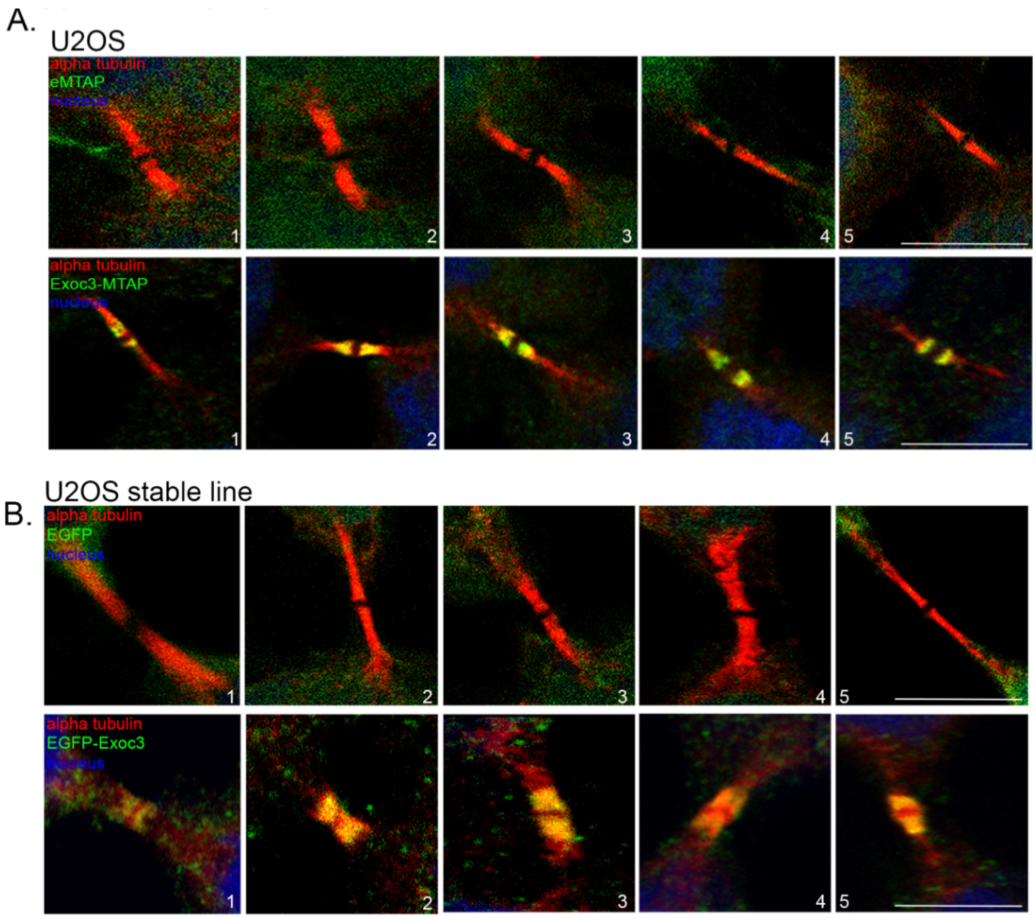

Figure S5: Exoc3 localization in the cytokinetic bridge and expression level of transgenic Exoc3-MTAP. A. Representative confocal micrographs of 5 independent cells in late cytokinesis of a U2OS cell line transfected with eMTAP (top) and Exoc3-MTAP (bottom). Green = eMTAP/ Exoc3-MTAP (immunostained with an anti-GFP antibody), red = tubulin (microtubules) stained using anti-tubulin antibody. Blue = DAPI (chromatin). Exoc3-MTAP localizes in the cytokinetic bridge at the regions flanking the midbody ring. The images are merged micrographs of all 3 fluorescence channels. **B.** Confocal micrographs of 5 U2OS cells stably expressing EGFP (top) or EGFP-Exoc3 (bottom). Green = EGFP/ EGFP-Exoc3 (immunostained with an anti-GFP antibody), red = tubulin (microtubules) stained using anti-α-tubulin antibody. Blue = DAPI (chromatin). EGFP-Exoc3 localizes in the cytokinetic bridge at the regions flanking the midbody ring. The images are merged micrographs of all 3 fluorescence channels.

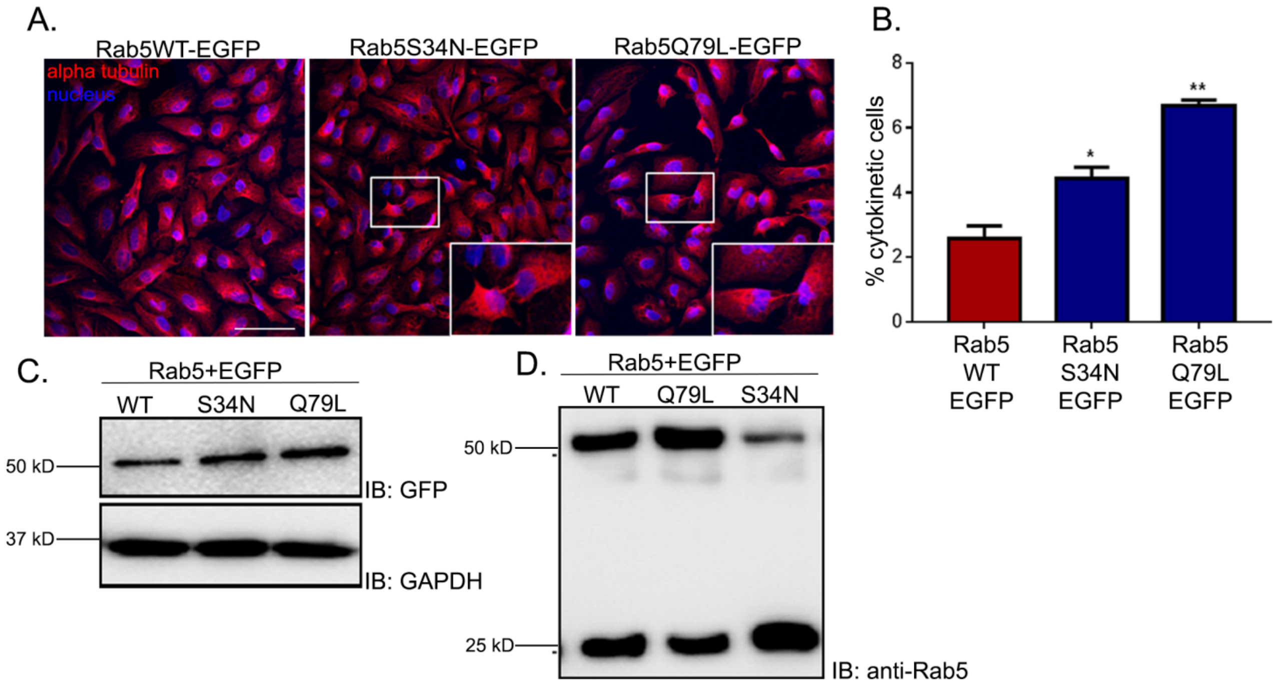

Figure S6: GTPase activity of Rab5 is required for cytokinesis. A. Representative fluorescence micrographs of HeLa cells transiently expressing Rab5 (WT, S34N and Q79L in the pEGFPC1 vector), fixed and stained for α tubulin (red) and chromatin (DAPI, blue). Scale bar: 75 μm, insets at bottom right show zoomed images of cytokinetic cells enclosed in small boxes. **B.** Quantification of percent cytokinetic cells from >500 cells over three independent experiments, expressed as mean ± S.D. (*P< 0.05). **C.** Immunoblot showing expression of the Rab5 constructs, GAPDH = loading control. **D.** Western blot showing relative expression levels of endogenous Rab5 and transgenic Rab5 mutants in HeLa cells.

Journal of Cell Science • Supplementary information

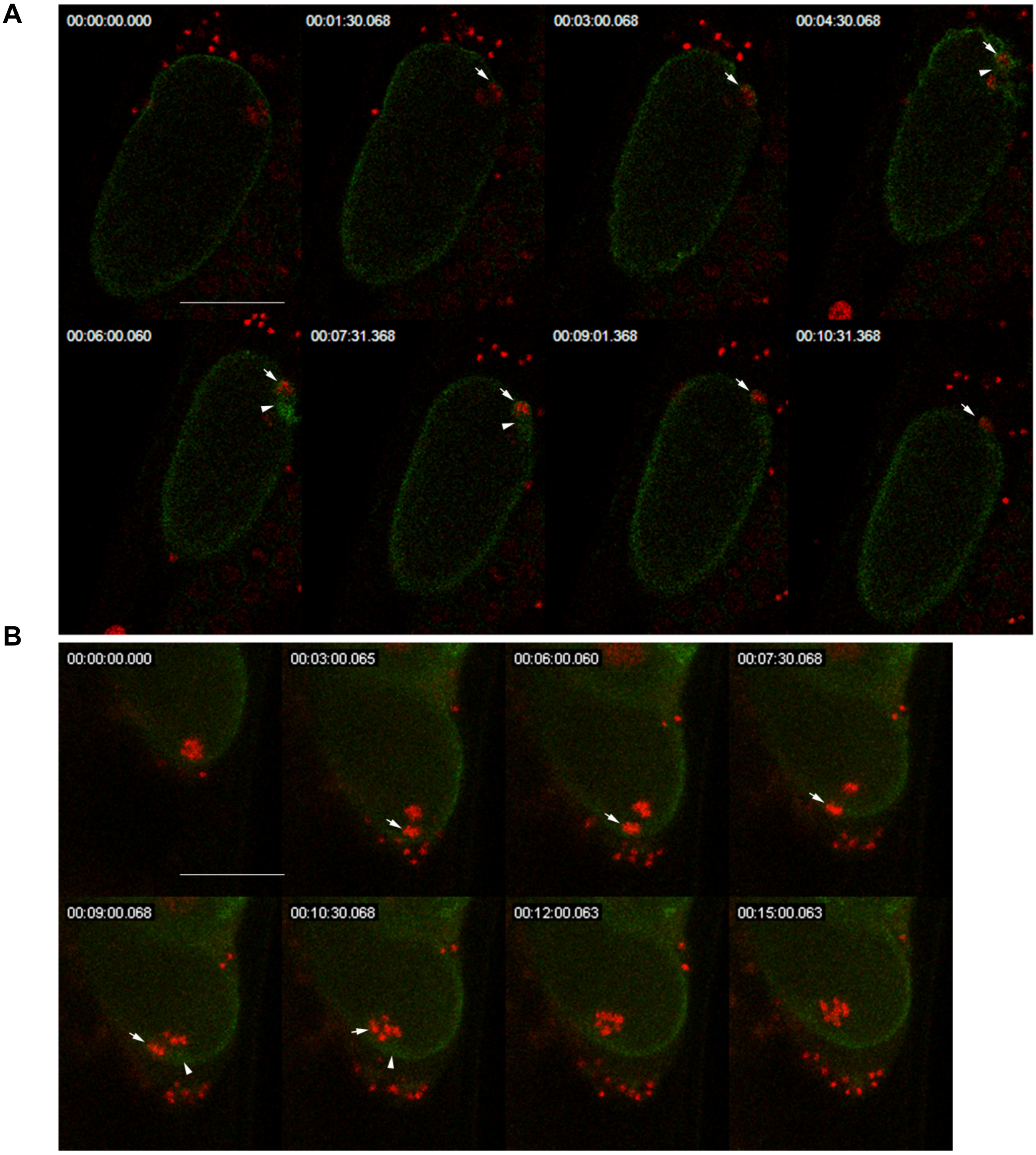

Figure S7: Sec-6 functions in polar body cytokinesis: Still images from the time-lapse videos of the first polar body cytokinesis (white arrow) in control (A) and sec-6 (RNAi) (B) worms. Membrane is marked by PH::GFP (green) and chromatin with mCherry::H2B (red). In control animals the polar body is successfully extruded out about 6 minutes post anaphase. However in the sec-6 (RNAi) worms the polar body is not extruded out even after 7-8 minutes post anaphase. Although the furrowing (shown by the white arrow) begins about 7 minutes post-anaphase, but it relapses back and the separated homologous chromosomes merge together, increasing the ploidy of the cell. Scale bar = 25µm.

Journal of Cell Science • Supplementary information

Table S1: Description of strains used in this study

Strain Name	Description	Source
N2	Bristol strain, Wild type	CGC
RT122	pwIs20 [pie-1p::GFP::rab-5 + unc-119(+)]	CGC
RT1043	pwIs403 [pie-1p::mCherry::rab-5 + unc-119(+)]	CGC
OD95	ltIs37[(pAA64) pie-1p::mCherry::his-58 + unc-119(+)] IV; ltIs38 [pie-1p::GFP::PH (PLC1delta1) + unc-119(+)]	CGC
OD70	ltIs44 [pie-1p::mCherry::PH(PLC1delta1) + unc-119(+)]	CGC
OD58	ltIs38 [pie-1p::GFP::PH(PLC1delta1) + unc-119(+)]	CGC
JJ1473	zuIs45 [nmy-2::NMY-2::GFP + unc-119(+)] V	CGC
VC2648	sec-8(ok2187) I/hT2 [bli-4(e937) let-?(q782) qIs48] (I;III)	CGC
tm4536	sec-6(tm4536) /mIn1 [mIs14 dpy-10(e128)] II	NBRP
MVS1	sec-6 (SMYL04[sec-6::GFP::SBP] II	This study
MVS2	pwIs20 [pie-1p::GFP::rab-5 + unc-119(+)]; ltIs44 [pie-1p::mCherry::PH(PLC1delta1) + unc-119(+)]	This study
MVS6	sec-6 (SMYL04[sec-6::GFP::SBP] II; ltIs44 [pie-1p::mCherry::PH (PLC1delta1) + unc-119(+)]	This study
MVS7	sec-6 (SMYL04[sec-6::GFP::SBP] II; pwIs403 [pie-1p::mCherry::rab-5 + unc-119(+)]	This study

Journal of Cell Science • Supplementary information

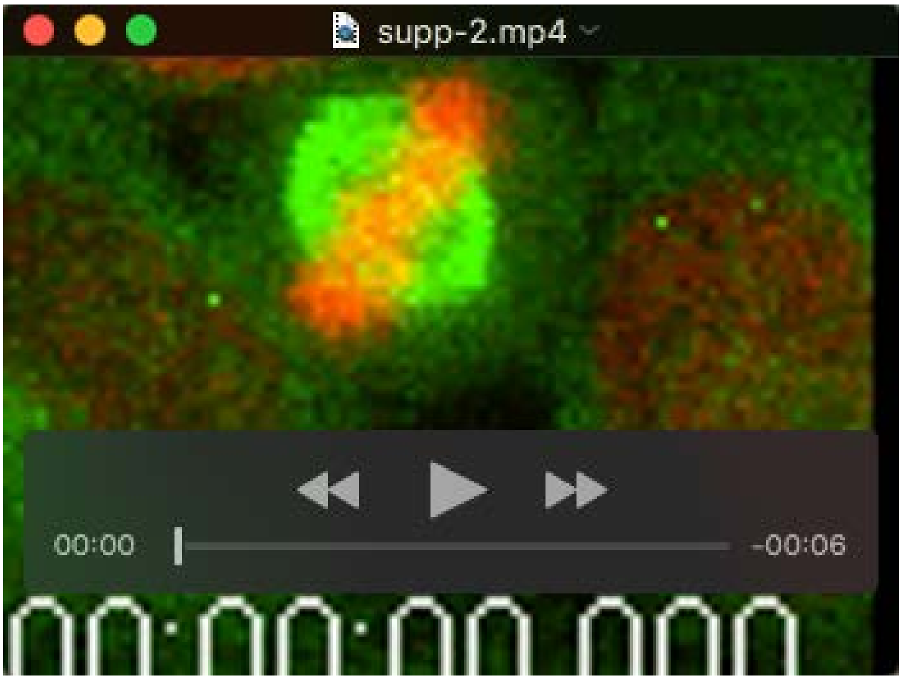

Movie 1: The video shows a control Luciferase siRNA transfected HeLa cell (stably expressing EGFP-α tubulin and mCherry-H2B) successfully completing cytokinesis post anaphase. Frames were captured every 3 min.

Journal of Cell Science • Supplementary information

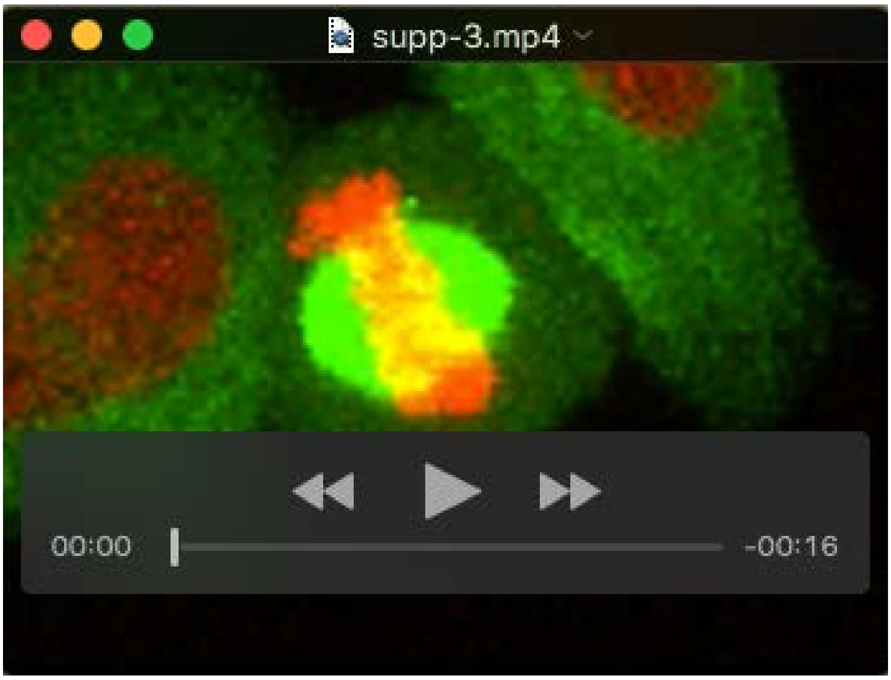

Movie 2: The video shows a HeLa cell with prolonged cytokinesis upon siRNA-mediated Exoc3 depletion. Frames were captured every 3 min.

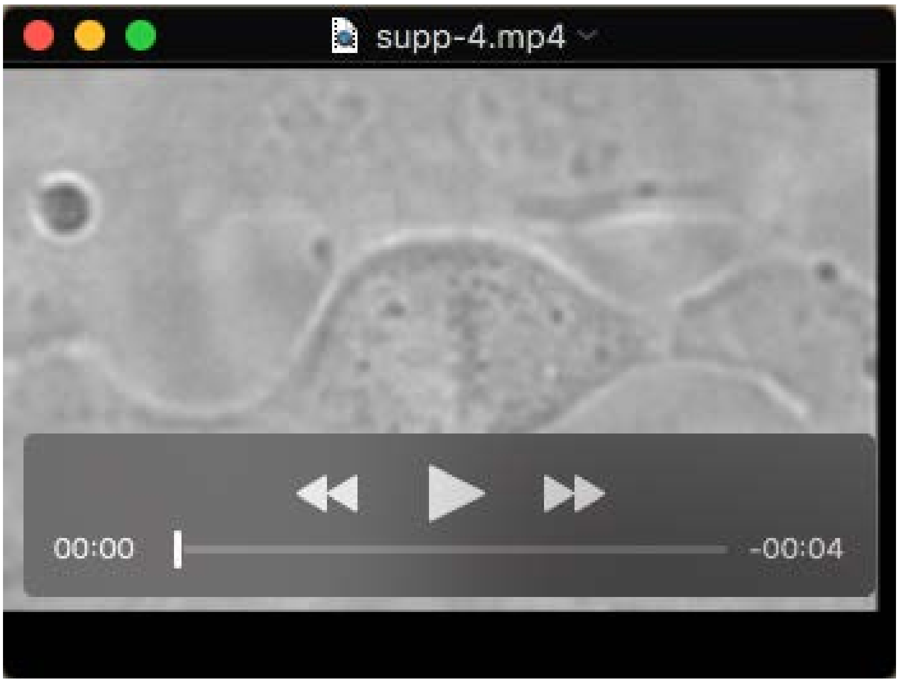

Movie 3: The video shows a control GFP siRNA transfected U2OS cell achieving successful abscission. Frames were captured every 5 min.

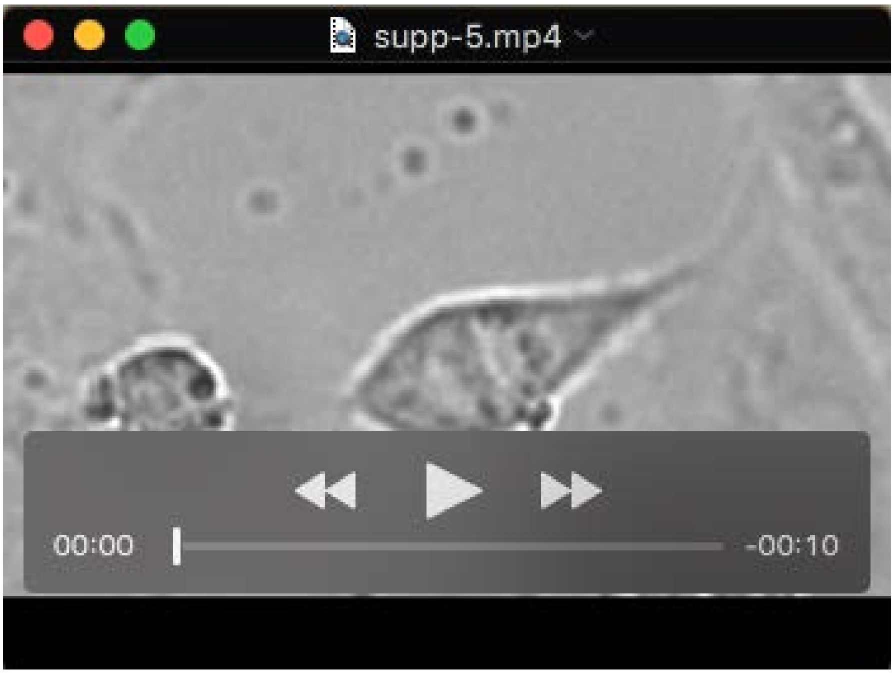

Movie 4: The video shows Exoc3 depleted U2OS cell experiencing delayed abscission. Frames were captured every 5min.

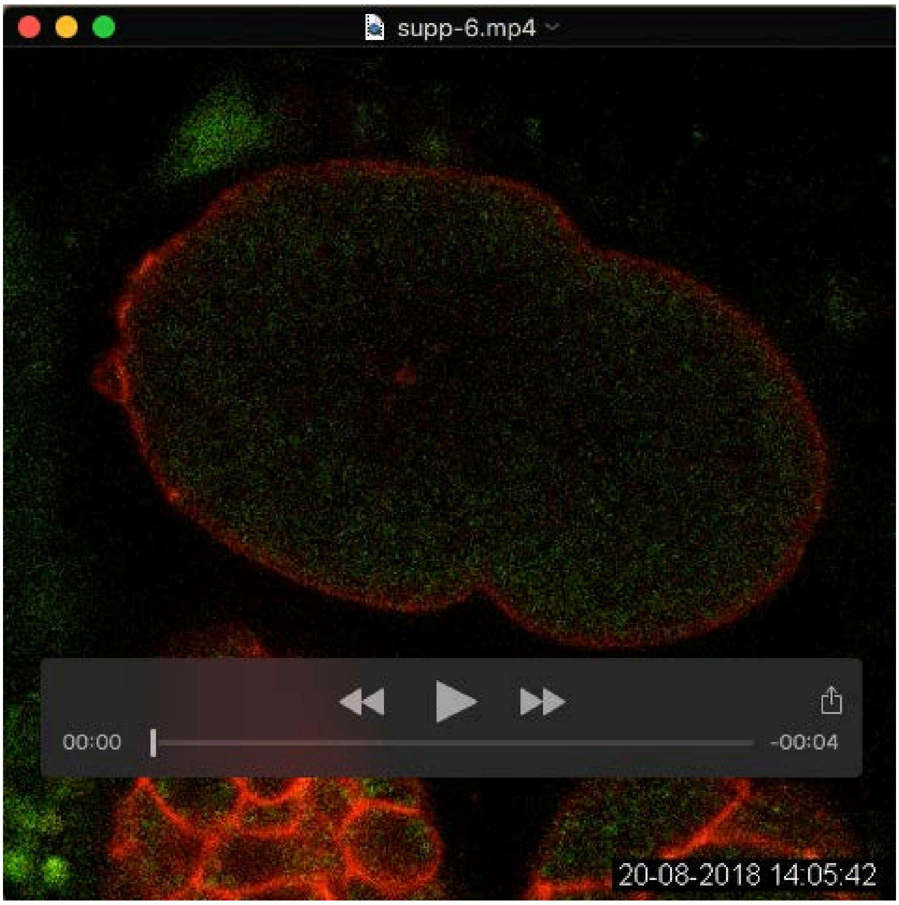

Movie 5: One cell stage *C. elegans* embryo expressing SEC-6::GFP and membrane marker PH::mCherry undergoing first division. Frames were captured every ~30s.

Journal of Cell Science • Supplementary information

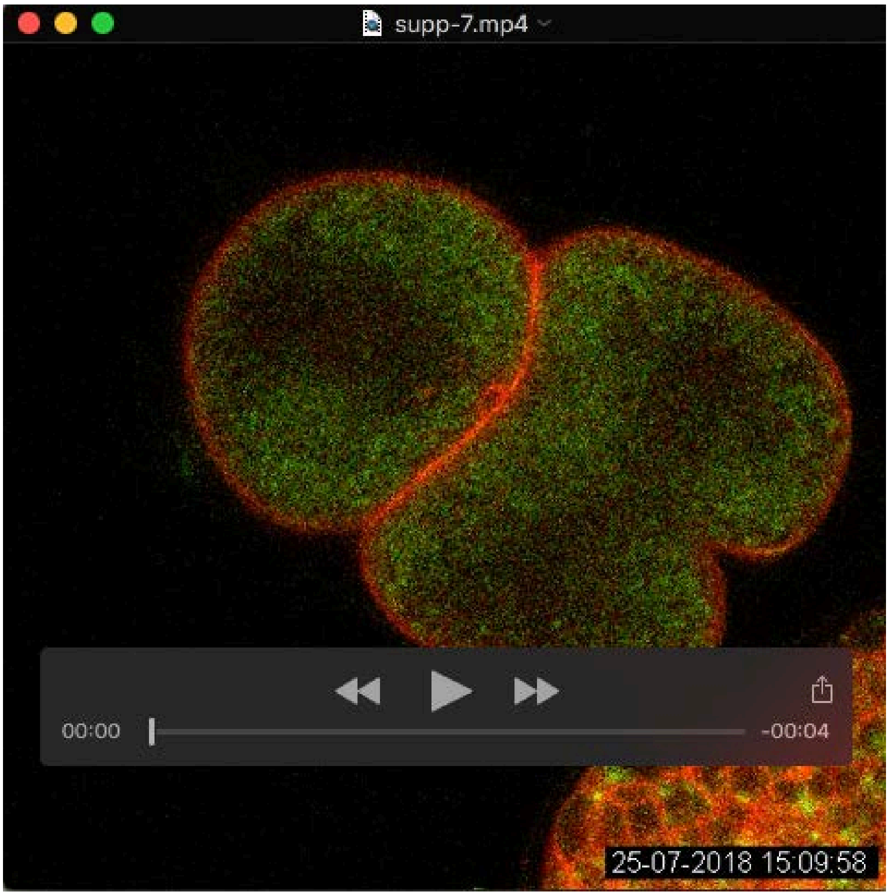

Movie 6: Two cell stage *C. elegans* embryo expressing SEC-6::GFP and membrane marker PH::mCherry undergoing the second division. Frames were captured every ~30s.

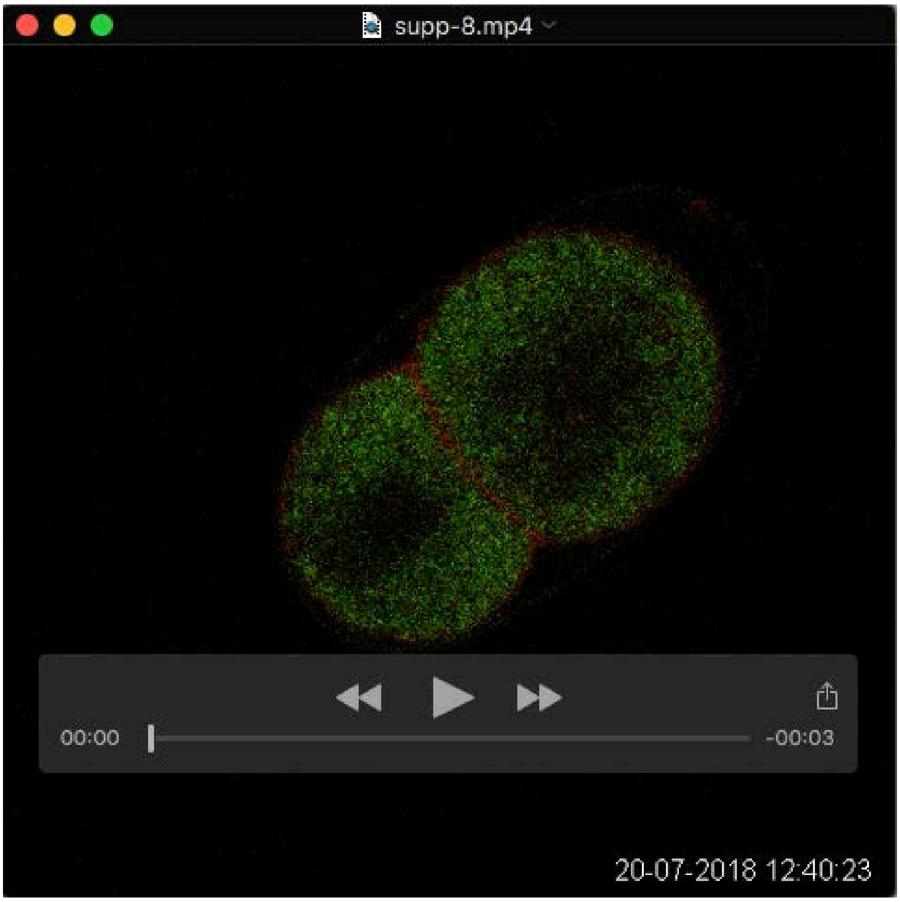

Movie 7: Two cell stage *C. elegans* embryo expressing RAB-5::GFP and membrane marker PH::mCherry undergoing the second division. Frames were captured every ~30s.

Journal of Cell Science • Supplementary information

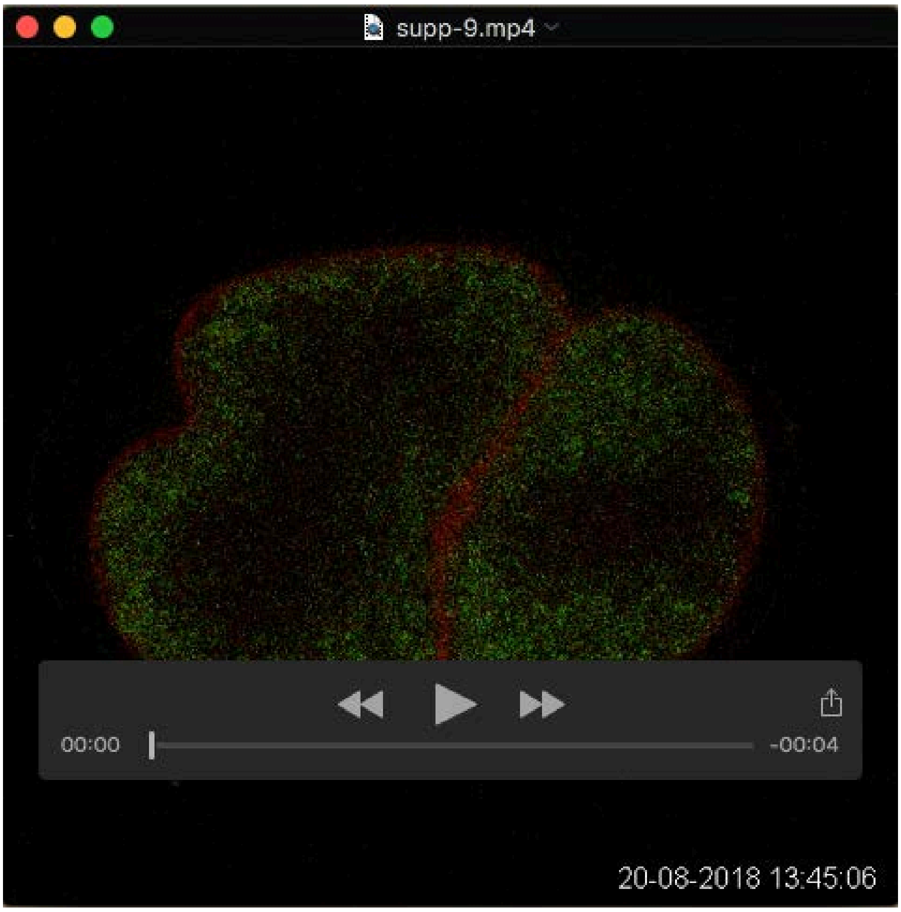

Movie 8: Two cell stage *C. elegans* embryo expressing RAB-5::GFP and membrane marker PH::mCherry undergoing the second and third divisions (between the 2-3 cell stage). Frames were captured every ~ 30s.

Journal of Cell Science • Supplementary information

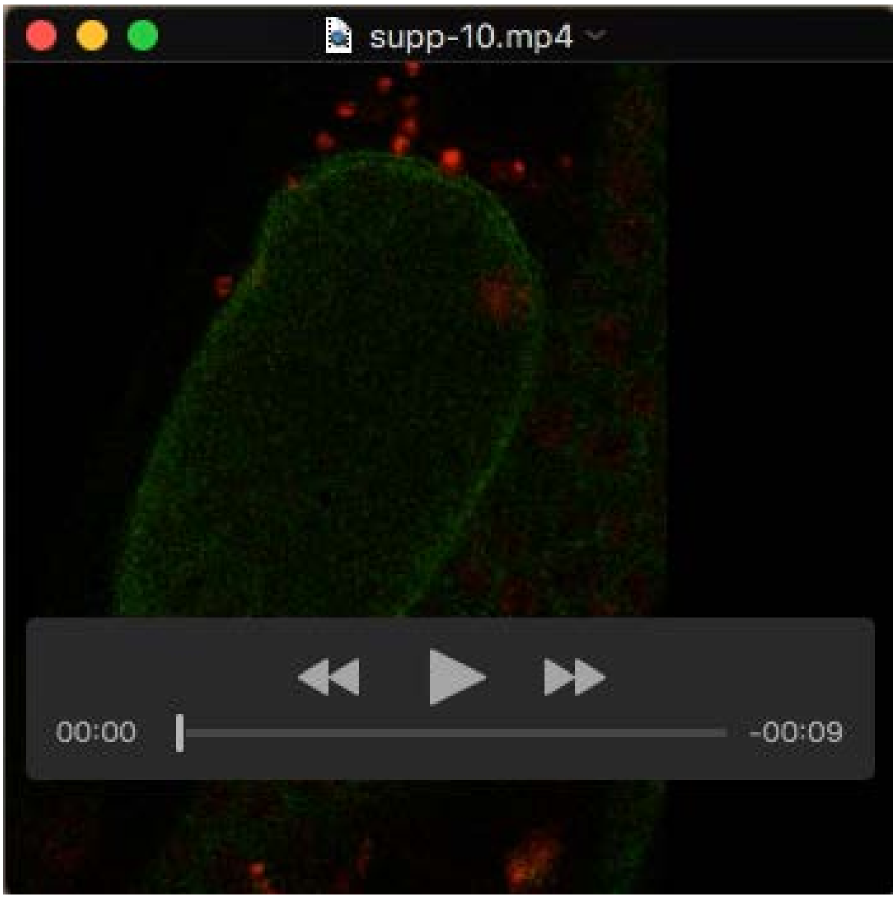

Movie 9: **Polar body cytokinesis in control RNAi worms:** Time lapse imaging showing first polar body extrusion in control *C. elegans* embryo imaged *in utero*. The polar body is successfully extruded out about 6 minutes post anaphase. Membrane is marked in green with PH::GFP and chromatin in red with mCherry::H2B.

Journal of Cell Science • Supplementary information

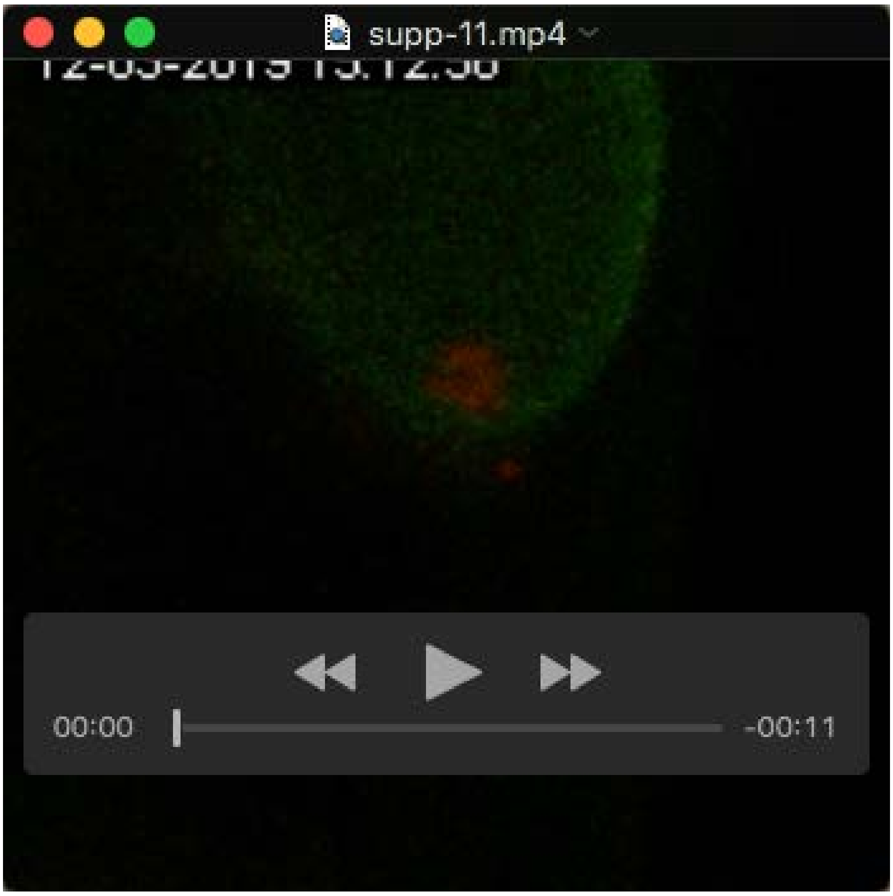

Movie 10: Polar body cytokinesis in *sec-6 (RNAi)* worms: Time lapse imaging showing first polar body extrusion in *sec-6 (RNAi) C. elegans* embryo imaged *in utero*. Polar body nucleus can be seen even after 8 minutes post-anaphase. Cellularization of polar body begins around this time with furrow ingression, but is not completed and finally relapses, leading to the internalization of the separated homologous chromosomes and increasing the ploidy of the embryo. Membrane is marked in green with PH::GFP and chromatin in red with mCherry::H2B.

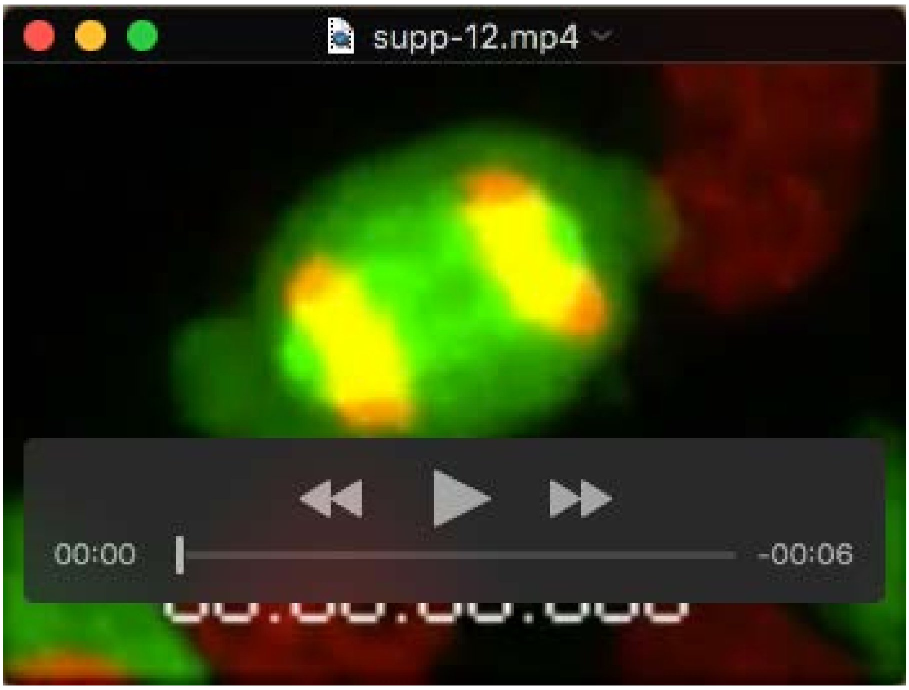

Movie 11: The video shows a control luciferase siRNA transfected HeLa cell (stably expressing EGFP-α tubulin and mCherry-H2B) successfully completing cytokinesis post anaphase. Successive frames were captured every 3 min.

Journal of Cell Science • Supplementary information

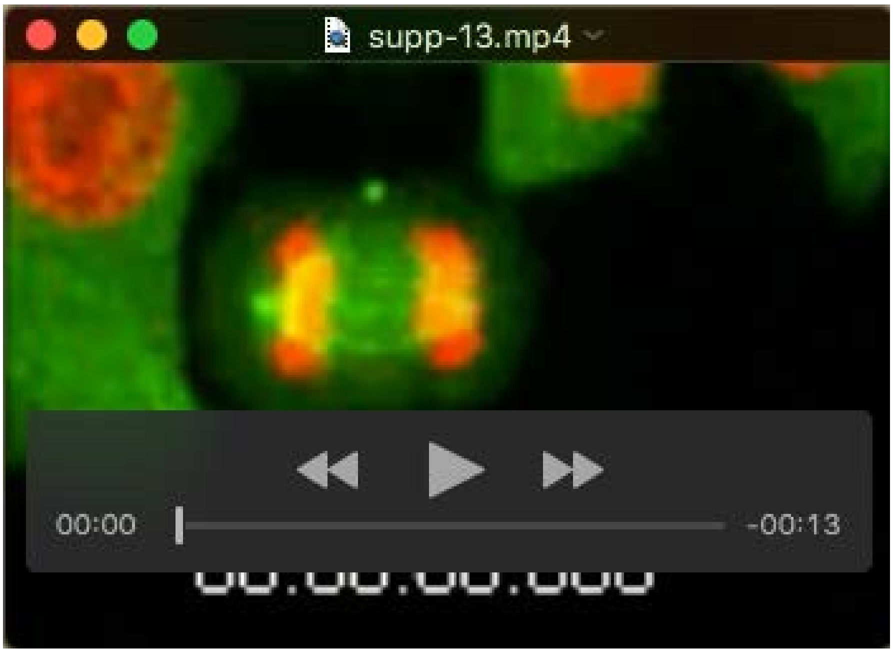

Movie 12: The video shows a HeLa cell exhibiting prolonged cytokinesis upon siRNA-mediated Rab5 depletion. Successive frames were captured every 3 min.
